# Efficacy of digital cognitive stimulation therapy for people with dementia: a systematic review and meta-analysis

**DOI:** 10.1186/s12877-026-07180-9

**Published:** 2026-04-06

**Authors:** Ita Daryanti Saragih, Pauline Hwang, Davis Yadav, Ling Qiu, Donna Marie Fick, S. Shyam Sundar, Saeed Abdullah

**Affiliations:** 1 College of Information Sciences and Technology, The Pennsylvania State University, Pennsylvania, USA; 2 Ross and Carol Nese College of Nursing, The Pennsylvania State University, Pennsylvania, PA USA; 3 Donald P. Bellisario College of Communications, The Pennsylvania State University, Pennsylvania, USA; 4 Department of Immersive Media Engineering, Sungkyunkwan University, Seoul, Republic of Korea

**Keywords:** Dementia, Digital health, Cognitive stimulation therapy, Meta-analysis

## Abstract

**Background:**

There is growing interest in using digital health technologies to deliver Cognitive Stimulation Therapy (CST) for people with dementia, given the potential to improve access and outcomes. However, evidence for the effectiveness of delivering CST using digital technologies (“digital CST”) remain limited, making it difficult to determine best practices. This study systematically reviews and evaluates the efficacy of digital CST on cognitive and psychosocial outcomes in people with dementia.

**Methods:**

We conducted a comprehensive literature search in CINAHL, Cochrane Library, Embase, MEDLINE, PubMed, and Web of Science. We included randomized controlled trials and quasi-experimental studies that examined the effects of digital CST in people with dementia. We assessed methodological quality using the Cochrane Risk of Bias tool (RoB 2) and ROBINS-I. We applied a DerSimonian and Laird random-effects model to estimate pooled standardized mean differences (SMDs).

**Results:**

Eighteen studies comprising 756 participants met inclusion criteria. Digital CST significantly improved cognitive function (k = 559; SMD = 0.33; 95% CI: 0.04–0.62; *p* = 0.03; I^2^ = 61.39%) and semantic fluency (k = 181; SMD = 0.43; 95% CI: 0.13–0.73; *p* < 0.001; I^2^ = 0.00%). It also significantly reduced depressive symptoms (k = 273; SMD = –0.66; 95% CI: –1.12 to –0.20; *p* = 0.01; I^2^ = 68.94%). The greatest cognitive improvements were observed when interventions were delivered biweekly in 45-minute sessions over fewer than eight weeks.

**Conclusions:**

This systematic review and meta-analysis demonstrates that digital CST is effective in enhancing cognitive performance and supporting aspects of psychosocial functioning in people with dementia. Improvements were observed particularly in global cognition, semantic fluency, and depressive symptoms, indicating that digital CST offers a beneficial non-pharmacological option within dementia care. Further research is warranted to explore its applicability across different dementia stages and to determine the sustainability of these effects over time.

**Prospero registration:**

CRD420250653224.

**Supplementary Information:**

The online version contains supplementary material available at 10.1186/s12877-026-07180-9.

## Background

Dementia is a global public-health priority, characterized by a progressive decline across multiple cognitive domains [1]. This deterioration profoundly impairs daily functioning and undermines psychological and social well-being [2, 3]. In 2021, more than 57 million people worldwide lived with dementia [4]. Projections suggest this figure will reach about 80 million by 2030 and 153 million by 2050, representing a twofold increase every twenty years [5, 6]. Despite these alarming trends, no curative treatment currently exists [4, 7]. Instead, available pharmacological options often produce adverse side effects [8], carry high financial costs [9, 10], remain inaccessible in many regions globally [11], and are approved only for select dementia subtypes primarily mild-to-moderate Alzheimer’s disease [12, 13]. These limitations underscore the need for incorporating evidence-based non-pharmacological interventions into standard care. Importantly, non-pharmacological approaches can be implemented safely over the long term, promoting cognitive preservation and improving the well-being of people with dementia [14, 15].

Among these interventions, Cognitive Stimulation Therapy (CST) has emerged as a structured psychosocial program specifically designed to activate key cognitive domains, including memory (both recent and past), language skills, and reasoning abilities [16]. CST sessions typically involve engaging in activities such as word categorization, associative word exercises, reminiscence discussions, and interactive games, all aimed at maintaining or enhancing cognitive functions [17, 18]. The theoretical underpinnings of CST are grounded in cognitive reserve theory and neuroplasticity principles, suggesting that sustained, structured cognitive stimulation can promote neural network reorganization and improve cognitive outcomes [19–21]. Central to its approach, CST is guided by 18 key principles that emphasize mental stimulation, the use of orientation, language stimulation, and a person-centered philosophy [22]. Through these principles, CST promotes communication and social interaction by incorporating structured group activities specifically designed to engage cognitive processes while simultaneously supporting interpersonal connection [23, 24]. This fosters meaningful engagement among participants, leading to enhanced social connections and reduced isolation. CST is the only non-pharmacological intervention currently recommended by the United Kingdom National Institute for Health and Care Excellence (NICE) guidelines for supporting and managing people with dementia [25, 26]. Given this strong evidence base and its multidimensional benefits, CST has emerged as a theoretically grounded and empirically supported intervention well-suited to meet the complex needs of people with dementia. Systematic reviews have consistently demonstrated that CST leads to positive outcomes for people with dementia, including improvements in cognitive function, quality of life, and neuropsychiatric symptoms [27–31]. These findings highlight the effectiveness of CST as a non-pharmacological intervention that not only targets cognitive enhancement but also addresses emotional connectivity and psychological well-being.

In the standard group CST protocol, sessions are designed for small groups of about 5–8 people [32, 33], delivered twice weekly, in a course of 14 sessions over seven weeks, with each session lasting around 45 minutes [30, 34]. However, in real-world practice, group size, session frequency, and session length can vary depending on the clinical setting [34]. To further accommodate individual needs, an adapted format known as individualized CST (iCST) is also available, offering a one-to-one delivery model [35]. While prior study reported low adherence and limited effectiveness when delivered by family carers [36], recent feasibility studies using professionally delivered and virtual iCST formats have demonstrated improved engagement and emerging cognitive benefits [37, 38]. A longer-term maintenance version (mCST) has also been developed, consisting of structured weekly sessions that have shown improvements in quality of life and potential cognitive gains, particularly when combined with pharmacological treatment [39]. However, despite its proven benefits, the routine implementation of CST remains challenging [40].

Delivering personalized, consistent sessions demands significant time and effort from healthcare providers and caregivers, often resulting in low adherence rates and reduced engagement among participants [41]. In response to these challenges, there has been a growing interest in the use of digital health technologies to facilitate CST delivery [38, 42]. Digital CST interventions can be delivered through several formats, including computer-based programs installed on desktops [43], web-based platforms accessed through internet browsers [44, 45], mobile applications that function online or offline [46], and videoconferencing tools such as Zoom that support real-time remote sessions [47]. Each format confers its own benefits, with app-based CST demonstrating improved accessibility and user engagement [46]. Likewise, video-conference delivery, as demonstrated by Spector et al. (2024), facilitates structured group sessions remotely and has shown promising feasibility and acceptability in dementia care [47]. By leveraging such technologies, it is possible to not only improve patient outcomes but also reduce the burden on healthcare systems [48, 49].

However, a successful transition to digital CST will need to address multiple challenges. One of the most significant obstacles is the limited familiarity with digital technologies among older adults and their care partners, which often leads to reluctance in adopting technology-based dementia interventions [50]. In addition to usability concerns, cost remains an important concern, as limited access to affordable devices, internet connectivity, and technical support continues to exclude many older adults from digital interventions [47, 51]. These financial barriers disproportionately affect vulnerable populations and risk deepening existing health disparities in dementia care [52]. Furthermore, research and development in digital CST remain fragmented, which has led to a lack of understanding regarding the strength and weakness of different strategies and approaches [53, 54]. Critical factors such as the characteristics of digital platforms, the optimal intervention dose, and the measurable outcomes remain insufficiently explored. While several review studies have explored the impact of digital CST interventions, the majority have not conducted comprehensive analyses of critical intervention parameters, such as platform characteristics, intervention dosage, and associated cognitive and psychosocial outcomes [55, 56]. Moreover, these reviews have often lacked systematic comparisons between different technological modalities, thereby limiting the ability to draw robust conclusions regarding their relative effectiveness and implementation fidelity. As a result, the current body of evidence provides a limited understanding of how people with dementia and their care partners perceive the usability, engagement, and benefits of digital CST platforms.

## Objective

The present study aims to systematically assess the effectiveness of digital CST interventions for people with dementia. The evaluation will focus specifically on outcomes related to cognitive performance and psychosocial functioning.

## Methods

We conducted the review in accordance with the Preferred Reporting Items for Systematic Reviews and Meta-Analyses (PRISMA) guidelines to ensure methodological transparency and reporting rigor (refer to Appendix A for details) [57].

### Literature search for identification of studies

We conducted a comprehensive literature search across seven electronic databases—CINAHL with Full Text, Cochrane Library, Embase, MEDLINE, PubMed, and Web of Science—from their inception through December 4, 2025. We developed search terms using Medical Subject Headings (MeSH) in CINAHL and PubMed, complemented by manual screening of terminology from relevant review articles [58–60]. The search strategy encompassed four primary conceptual domains: (1) dementia-related conditions (e.g., dementia, Alzheimer’s disease, Lewy body dementia, Parkinson’s disease dementia, frontotemporal dementia, mixed dementia, vascular dementia) [24, 37, 59]; (2) any form of cognitive stimulation therapy (e.g., cognitive stimulation therapy [CST], individual CST [iCST], maintenance CST [MCST], cognitive interventions, memory therapy, memory groups, reality orientation, cognitive psychostimulatory) [24, 37, 58, 59]; (3) digital health technologies (e.g., digital health, eHealth and computer-based and web-based interventions, mobile applications, wearable devices, videoconferencing tools, virtual and augmented reality systems) [60]; and (4) experimental study designs (e.g., randomized controlled trials, quasi-experimental studies, pretest-posttest designs, and other interventional methodologies). Additionally, we performed a manual search of grey literature via Google Scholar and by examining the reference lists of relevant systematic reviews [55, 56]. A detailed account of the search strategy is available in Appendix B.

### Eligibility criteria

We considered studies eligible if they met the following Population, Intervention, Comparison, Outcomes, and Study design (PICOS) criteria. The inclusion criteria were as follows:

- (P) Population included individuals diagnosed with any type of dementia, such as Alzheimer’s disease, vascular dementia, frontotemporal dementia, mixed dementia, or other forms, without restrictions on sex, living situation, or disease severity.
- (I) The intervention involved any form of digital-based cognitive stimulation therapy.
- (C) The comparison group received standard care or traditional cognitive stimulation programs delivered through non-digital means (e.g., in-person CST sessions), reflecting the conventional format used in most clinical and community settings.
- (O) Outcomes included cognitive function, psychological well-being (e.g., mood, depression, or anxiety), or quality of life.
- (S) Design of peer-reviewed randomized controlled trials, non-randomized studies, and dissertations that provided sufficient pre- and post-intervention data, including means and standard deviations for both intervention and control groups, to enable the calculation of effect sizes.

No language restrictions were applied. Studies were excluded if they were protocols, case reports, review articles, unpublished reports, conference abstracts, or if they lacked the necessary data to compute mean differences or standardized mean differences (SMD) for the outcomes of interest.

### Study selection and data extraction

Two reviewers (IDS and PH) independently conducted the study selection process using the Covidence platform, in accordance with PRISMA guidelines [61]. IDS imported all retrieved records into Covidence, where automatic duplicate removal was applied. During the initial screening phase, both reviewers independently reviewed titles and abstracts to identify potentially eligible studies. Afterwards, full-text articles were assessed using the same independent procedure, but only for those selected based on their titles and abstracts**.** Any disagreements regarding study inclusion were resolved through discussion with SA to reach a consensus. Following the selection process, IDS extracted key data from the included studies, with PH verifying the accuracy and completeness of the extracted data. Data were extracted directly from the authors’ descriptions in the published articles, and no quotations were used. Extracted variables included: country where the intervention was conducted, study design, total number of participants in the intervention and control groups, intervention provider, mean age of participants in both groups, type of residential setting, type and severity of dementia, and type of digital platform used for delivering CST. We also extracted intervention characteristics, including intervention modality, level of supervision, mode of delivery, type of digital CST, and intervention dosage (session duration, frequency per week, hours per week, and total duration). Additionally, we collected data on the presence of follow-up, type of control intervention, and the mean and standard deviation (SD) of reported outcomes along with their measurement instruments.

### Quality assessment of the studies included and GRADE evidence

Two reviewers (IDS and DY) independently assessed methodological quality of the included studies. For randomized controlled trials, Version 2 of the Cochrane Risk of Bias tool (RoB 2), as recommended by Cochrane Reviews, was employed [62]. This tool evaluates five domains of potential bias: the randomization process, deviations from intended interventions, missing outcome data, measurement of outcomes, and selection of the reported results [63]. Each domain was rated as having a low risk, some concerns, or high risk of bias [64]. We excluded studies with a high risk of bias in three or more domains from the meta-analysis due to concerns that such bias could significantly distort the pooled effect estimates.

For non-randomized studies, we used the Risk Of Bias In Non-randomized Studies - of Interventions (ROBINS-I) tool [65, 66], which assesses seven domains of bias: confounding, selection of participants, classification of interventions, deviations from intended interventions, missing data, measurement of outcomes, and selection of the reported result [65]. Each domain was rated as low, moderate, serious, or critical risk of bias [67]. We excluded studies with a critical risk of bias in four or more domains from the meta-analysis, as such bias was deemed likely to compromise the validity of the pooled results.

In addition, we used the GRADE (Grading of Recommendations, Assessment, Development and Evaluation) approach to evaluate the overall certainty of the evidence for each pooled SMD [68, 69]. Any disagreements regarding study inclusion were resolved through discussion with SA to reach a consensus. The certainty of evidence was assessed across five domains: risk of bias, inconsistency, indirectness, imprecision, and publication bias [69, 70]. Based on these criteria, the quality of evidence was categorized as high, moderate, low, or very low allowing for a transparent appraisal of the strength and certainty of the findings [71].

### Statistical analysis

We conducted meta-analysis using STATA version 17.0 (StataCorp LLC, 4905 Lakeway Drive, College Station, Texas, USA), based on the pre-post mean and SD values from both intervention and control groups. We calculated mean changes and corresponding raw SDs for each group to compute the SMD, or Cohen’s d, with 95% confidence intervals (CI) [72, 73]. We chose SMD as the effect size metric due to the variation in outcome measurement tools across studies for cognitive function, semantic fluency, depressive symptoms, and quality of life. Given the limited number of studies included in each outcome analysis (fewer than 10), we used a DerSimonian and Laird random-effects model to estimate pooled effect sizes [74, 75]. We assessed heterogeneity among studies using the Q and *I*^*2*^ statistic, with values greater than 50% indicating substantial heterogeneity. We also generated Forest plots to visualize the meta-analytic results [76]. Where ten or more studies were included in an analysis, potential publication bias was assessed using funnel plots [77] and Egger’s regression test [78]. In addition, we performed a leave-one-out sensitivity analysis for each pooled SMD to evaluate the robustness of the results by iteratively removing the study with the greatest weight in the analysis [74].

## Results

### Study selection

**Fig. 1:**
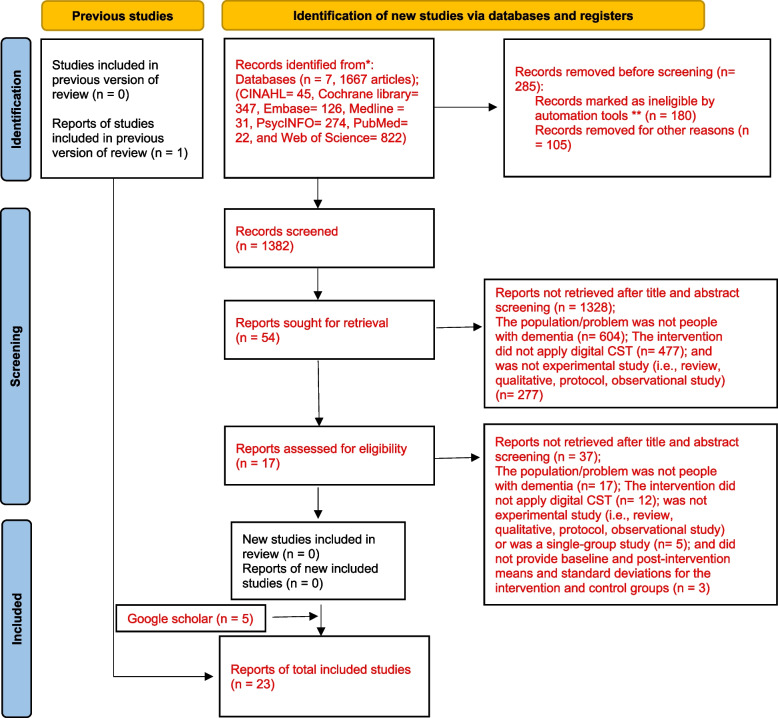
PRISMA flowchart diagram. *Consider, if feasible to do so, reporting the number of records identified from each database or register searched (rather than the total number across all databases/registers). **If automation tools were used, indicate how many records were excluded by a human and how many were excluded by automation tools. *From:* Page MJ, McKenzie JE, Bossuyt PM, Boutron I, Hoffmann TC, Mulrow CD, et al. The PRISMA 2020 statement: an updated guideline for reporting systematic reviews. BMJ 2021;372:n71. https://doi.org/10.1136/bmj.n71

The database search initially yielded 1,667 records, of which 285 were identified as duplicates and removed. We screened the remaining 1,382 records based on their titles and abstracts using the PICOS framework. Of these, we excluded 1,328 articles for the following reasons: the population did not include people with dementia (k = 604); the intervention did not involve digital CST (k = 477); or the study design was not experimental (e.g., review articles, qualitative studies, protocols, or observational studies) (k = 277). This screening process resulted in 54 articles being selected for full-text review. Upon further evaluation, 37 articles were excluded due to the following reasons, which were not detected in the earlier screening: the population was not individuals with dementia (k = 17); the intervention did not utilize digital CST (k = 12); the study was not an experimental design (k = 5); the study did not provide baseline and post-intervention means and standard deviations for both the intervention and control groups (k = 3). Ultimately, we identified 17 studies through database searches. An additional five studies were identified through a Google Scholar search, and one more was retrieved through manual searching of references from relevant review articles. In total, 23 studies were included in the final analysis [38, 47, 79–99]. The detailed study selection process is shown in Fig. 1.

### Study characteristics

This review included 18 studies conducted across Austria [99], Australia [80], Brazil [84], China [97], India [79], Iran [92], Italy [81, 85, 93], the Netherlands [83], Portugal [89, 94, 95], South Korea [88], Spain [82, 86, 96], Turkey [90], the United Kingdom [38, 47, 91], and the United States [87, 98], comprising 20 randomized controlled trials and 3 quasi-experimental studies. Across all studies, a total of 1,002 participants with mild to severe dementia were enrolled (k = 556 in the intervention group and k = 436 in the control group). Participants were drawn from a range of living arrangements and care settings, including nursing homes, residential care facilities, day centers, home environments, outpatient memory clinics, and community care centers. The mean age of participants in the intervention groups ranged from 71.9 years (SD = 9.2) to 86.0 years (SD = 5.2), and from 70.8 years (SD = 9.3) to 85.6 years (SD = 10.0) in the control groups.

The digital CST interventions were delivered through a range of platforms, such as mobile applications, computer-based program, videoconferencing, virtual reality, and web-based. Supervision models varied, including remote-only, face-to-face, and hybrid delivery approaches. The content of the CST programs included a variety of cognitively engaging tasks such as musical and audiovisual memory prompts (e.g., Memory Box), life-stage photo reminiscence, interactive games, short narrative-based questions, sensory stimulus tasks, autobiographical recall, social problem-solving, planning of everyday activities, and analysis of personal schedules.

Intervention sessions typically lasted 30 to 60 minutes and were delivered between one and five times per week. Control conditions included usual care, traditional paper-and-pencil CST, or standard cognitive training. These sessions were led by professionals such as psychologists, occupational therapists, neuropsychologists, neurologists, cognitive therapists, trained research assistants, trained volunteers, nurses, and speech and language therapists. The duration of interventions ranged from 4 to 12 weeks, with the cumulative dose of therapy ranging from 2 to 36 total hours. Outcomes were assessed at baseline and immediately post-intervention, with some studies also including a follow-up assessment up to 24 weeks post-intervention to examine the persistence of effects. A summary of the included studies and their characteristics is presented in Table 1.

**Table 1:** Summary of included studies

**No**	**Citation/Country**	**Study design**			**Participants characteristics**				**Intervention characteristics**										**Control group**	**Outcome (tools)**
			**Sample size (n), (i/c)**	**Intervention provider**	**Mean (SD) of age (i/c)**	**Living environment**	**Type of dementia**	**Severity of dementia**	**Digital-based type**	**Intervention modality**	**Supervision type**	**CST delivery**	**Digital CST group**	**Session length (min)**	**Frequency per week**	**Dose (h)**	**Duration (weeks)**	**Follow-up**		
1	Bhowmik et al., 2023/India [79]	RCT	57, 27/30	Clinical psychologist/counselor	66.1(8.1)/68.3(9.3)	Hospital	Alzheimer’s disease, vascular dementia (VD), mixed dementia, frontotemporal dementia and Lewy body dementia	NA	Videoconferencing using WhatsApp CST groups/video calling/telephonic interactions/Google meet/zoom platforms	Remote	Supervised	Group	Manual-based group therapy adapted and validated for the north Indian population	45	1	10.5	7	Baseline and post-intervention	Usual care	Neuropsychiatric symptoms (NPI): Delusions, hallucinations, agitation, depression, anxiety, euphoria, apathy, disinhibition, irritability, motor disturbance, nighttime behaviors, appetite, caregiver burden (ZBI), cognitive function (ADAS cog)
2	Davison et al., 2016/Australia [80]	RCT	9, 6/3	Trained research assistant	86.0(5.2)	Nursing home	Dementia	Mild to severe	Computer-based program	In-person	Supervised	Individual	Memory box: music, movies, messages and photos	30	1	2	4	Baseline and post-intervention	Social interaction	Agitation (CMAI), depression symptom (CSDD), anxiety (RAID)
3	De Luca et al., 2016/Italy [81]	RCT	20, 10/10	Neuropsychologist, neurologist andcognitive therapist	78.0(5.4)/77.8(5.3)	Nursing home	Mixed dementia and vascular dementia	Mild to moderate	Web-based	NA	NA	NA	A program with a series of seven pc-based activities to stimulate cognitive domains	45	3	18	8	Baseline and post-intervention	Cognitive therapy using a paper-and-pencil	Cognitive function (MMSE), verbal fluency (CVF), attention (AM), daily activity (ADL), instrumental daily activity (IADL), dementia severity (BANSS), depression (GDS)
4	Domenech et al., 2023/Spain [82]	RCT	31, 16/15	Psychologist	85.0(8.0)/84.0(8.0)	Residential care	Dementia	NA	Application	Remote	NA	Individual	Memory of childhood, adolescence, and adulthood using photographs	45	1	3.75	5	Baseline and post-intervention	Memory of childhood, adolescence, and adulthood using photographs delivered by psychologist	Depression symptom (CSDD), communication ability (HCS), cognitive function (MMSE), quality of life (QoL-AD)
5	Elfrink et al., 2021/Netherlands [83]	RCT	42, 23/19	Trained volunteer	79.5(8.1)/81.2(11.2)	Home	Dementia	Mild	e-health application	In-person	Supervised	Individual	Personal memory	NA	5	NA	8 to 10	Baseline, 3 months, and 6 months	Usual care	Neuropsychiatric symptoms (NPI), apathy (NA), caregiver's quality of life (CarerQol), caregiver’s life satisfaction (RAND-36), caregiver distress (NPI)
6	Fernández-Calvo et al., 2011/Brazil [84]	Quasi-experimental study with a pretest–posttest design	45, 30/15	Occupational therapist and psychologist	75.8(4.3)/75.6(4.6)	NA	Alzheimer's disease	Mild	Computer-based program	In-person	Supervised	Individual	Psychostimulation games	60	3	36	12	Baseline and post-intervention	Traditional stimulation program based on paper-and-pencil tasks	Cognitive function (ADAS-Cog), neuropsychiatric symptoms (NPI), depression (CSDD), disability (RDRS-2)
7	Galante et al., 2007/Italy [85]	RCT	11, 7/4	Neuropsychologist	76.0(6.0)	NA	Dementia	Mild	Computer-based program	In-person	Supervised	Individual	Patient’s life history, hobbies and favorite activities	60	3	3	4	Baseline, post-intervention, and 3 months	Sessions of semi-structured interviews	Cognitive function (MMSE and MODA), word repetition, verbal fluency, Neuropsychiatric symptoms (NPI), daily activity (BADL), instrumental daily activity (IADL), depression (GDS)
8	Gonzalez-Moreno et al., 2022/Spain [86]	Quasi-experimental with a control and treatment group	98, 50/48	Therapist	NA	Care center	Dementia	Moderate	Computer-based program	In-person	Supervised	Individual	Short story questions, stimulus selection, social problem solving, recall of biographical moments, planning of simpletasks, and schedule analysis	50	2	13.3	8	Baseline and post-intervention	Usual care	Depression (GDS), cognitive function (MMSE), semantic fluency (BT)
9	Hui et al., 2024/United Kingdom [38]	RCT	34, 17/17	Therapist	70.3(8.5)/72.9(8.9)	Home	Dementia	Mild to moderate	Videoconferencing using Zoom	Remote	Supervised	Individual	Physical games, life story activities, sounds-based stimulation, childhood-related prompts, food-related activities, faces and scenes, word association, creative activities, categorizing objects, orientation tasks, using money, number games, word games, and thinking cards.	45	2	10.5	7	Baseline and 9 weeks	Usual care	Cognitive function (ADAS-Cog and MOCA-BLIND), quality of life (QoL-AD), depression (GDS-15), Communication (HCS)
10	Marin et al., 2022/United States [87]	RCT	19, 10/9	Research staff	72.5(6.0)/75.0(13.0)	Home	Alzheimer's disease	Mild	Application	Remote	Unsupervised	Individual	Cognitive exercises for stabilizing language, attention, and memory functioning	30	1	12	24	Baseline, 6 weeks, 12 weeks, 18 weeks, and 24 weeks	Booklets of puzzles and brain teasers	Adherence, constant therapy usage, task performance
11	Moon & Park, 2020/South Korea [88]	RCT	49, 25/24	Nurse	82.9(6.0)/84.1(6.2)	Daycare	Alzheimer's disease, vascular dementia, and other	Moderate	Application	In-person + remote	Supervised	Individual	Reminiscence therapy	30	2	8	8	Baseline, post-intervention, and 4 weeks post-intervention	Storytelling without digital materials	Cognitive function (MMSE), depression (CSDD), Behavioral and psychological symptoms of dementia (NPI), engagement (EPWDS)
12	Oliveira et al., 2021/Portugal [89]	RCT	17, 10/7	Neuropsychologist	82.6(5.4)/84.1(6.3)	Residential care home	Alzheimer's disease	Mild to moderate	Virtual reality	In-person	Supervised	Individual	System Lisbon battery including morning hygiene, the shoe closet test, the wardrobe test, a memory test, the virtual kitchen, the TV news task, the grocery store task, the pharmacy task, and the art gallery test.	45	2	9	4 to 6	Baseline and post-intervention	Usual care	Executive function (FAB), cognitive function (MMSE), instrumental daily activity (IADL)
13	Parlak et al., 2024/Turkey [90]	RCT	32, 16/16	Speech and language therapists	75.0(6.4)/74.6(6.6)	Outpatient clinic	Alzheimer's disease	Mild to severe	Application	In-person + remote	Supervised	Individual	Consensus questions for emotion, cognition, and communication	30	2	7	7	Baseline and post-intervention	Pharmacological treatment	Cognitive function: orientation, attention and calculation, recall, language, and total score (MMSE), speech fluency, auditory comprehension, repetition, naming, and total score (LATA)
14	Rai et al., 2020/United Kingdom [91]	RCT	61, 31/30	Caregiver	74.4(6.8)/71.8(8.5)	Primary and secondary care, memory clinics, support groups, Join Dementia Research (JDR: an online register), and social media	Dementia	Mild to moderate	Application	Remote	Supervised	Individual	iCST program incorporated a mix of game-like and active features, combining audio-visual stimuli with structured discussion questions	30	3	16.5	11	Baseline and post-intervention	Usual care	People with dementia: cognitive function (ADAS-Cog), quality of life (QoL-AD), depression (CSDD), daily activity (BADLS), neuropsychiatric symptoms (NPI); Carer: quality of life (EQ-5D), anxiety (HADS), depression (HADS), carer-patients relationship (QCPR)
15	Sedigh et al., 2024/Iran [92]	Quasi-experimental study with a pretest–posttest design	42, 21/21	Caregiver	NA	NA	Alzheimer's disease	Mild to moderate	Application	Remote	Supervised	Individual	Numerical memory task	45	2	6	4	Baseline and post-intervention	No treatment provided	Mental status, cognitive function: time awareness, location awareness, record and recall, attention and calculation, recent memory, different language functions, spatial thinking (MMSE)
16	Serino et al., 2017/Italy [93]	Non-RCT	20, 10/10	Neuropsychologist	NA	Senior center	Alzheimer's disease	NA	SenseCam, a wearable digital camera	In-person	Supervised	Individual	Memory task on objects	NA	3	NA	3 to 4	Baseline and post-intervention	Conventional cognitive training	Cognitive function (MMSE), verbal fluency test, verbal categorical test, executive function (FAB), short-term memory abilities (Digit Span Test)
17	Silva et al., 2017a/Portugal [94]	RCT	51, 34/17	Neuropsychologist	75.4(5.3)/71.7(.2)/	Daycare	Alzheimer's disease	Mild	SenseCam, a wearable digital camera	In-person	Supervised	Individual	Memory stimulation via images	60	2	12	6	Baseline, 1-week post-intervention, and 6 months post-intervention	Cognitive therapy using a paper-and-pencil	Short-term memory abilities (Digit Span Test), verbal fluency (NA)
18	Silva et al., 2017b/Portugal [95]	RCT	46, 31/15	Neuropsychologist	75.4(5.3)/71.7(.2)/	Daycare	Alzheimer's disease	Mild	Computer-based program	In-person	Supervised	Individual	Memory stimulation via images	60	2	12	6	Baseline, 1-week post-intervention, and 6 months post-intervention	Cognitive therapy using a paper-and-pencil	Depression symptom (GDS), functional capacity (IAFAI), quality of life (WHOQOL-OLD)
19	Spector et al., 2024/United Kingdom [47]	RCT	46, 24/22	NA	71.9(9.2)/70.8(9.3)	London Memory Services Network Group, Memory-Matters, Age UK, Camden Carers, and the Join Dementia Research network (JDR, an online recruitment platform	Dementia	Mild to moderate	Videoconferencing using Zoom	Remote	Supervised	Group	14-session themes of CST (e.g., “physical games,” “being creative,” “childhood,” “food,” and “current affairs”)	60	2	14	7	Baseline and post-intervention	Treatment as usual	Cognitive function (MOCA-BLIND and ADAS-Cog), depression symptom (GDS), quality of life (QoL-AD
20	Tárraga et al., 2006/Spain [96]	RCT	43, 31/12	NA	75.8(5.9)/77.4(4.7)	Clinic and daycare	Alzheimer's disease	Mild	Web-based	In-person	NA	NA	Memory stimulation exercises across the domains of attention, calculation, language, memory and orientation	20	3	8	12	Baseline, week 12, and week 24	Cognitive stimulation tasks, workshops, and reinforcement of instrumental activities of daily living	Cognitive function (MMSE and ADAS-cog), naming ability (BNT), semantic fluency, story recall (RBMT)
21	Wong, 2024/China [97]	RCT	124, 61/63	NA		Elderly center	Dementia	Mild to moderate	Videoconferencing	Remote	Supervised	Group	Virtual CST protocol adapted to Hong Kong Chinese culture	NA	1	NA	7	Baseline and post-intervention	in-person CST protocol adapted to Hong Kong Chinese culture	Cognitive function (ADAS-Cog), quality of life (QoL-AD), social function (HKSF-DEM)
22	Yu et al., 2019/United States [98]	RCT	80, 64/16	NA	80.7(7.0)/85.6(10.0)	Senior facilities and community center	Dementia	NA	Application	In-person	Supervised	Group + individual	Games	30	2	6	6	Baseline, 6 weeks, and 12 weeks	Usual care	Quality of life (QoL ladder), mood (AMS), behavioral and psychological symptoms of dementia (NPI), caregiver distress (NPI)
23	Zuschnegg et al., 2025/Austria [99]	RCT	25, 12/13	Professional trainer	76.6(9.2)/74.9(7.0)	Home	Alzheimer's disease	Mild to moderate	Tablet-based - Application	Remote	Supervised	Individual	Cognition, movement, perception, ADLs, games and creativity	NA	NA	NA	24	Baseline and post-intervention	Paper-and-pencil cognitive exercises	Cognitive function (MMSE and MoCA), verbal pairs (WMS-R), word fluency (VLMT)

*NA* Not Available, *RCT* Randomized Controlled trial, *ADAS-Cog* Alzheimer's Disease Assessment Scale-Cognitive, *ADL* Activities of Daily Living, *AM* Attentive Matrices, *BADL* Bayer Activities of Daily Living Scale, *BADLS* Bristol Activities of Daily Living Scale, *BANSS* Bedford Alzheimer Nursing Severity Scale, *BNT* Boston Naming Test, *BT* Barcelona test, *CarerQoL* Care-related Quality of Life instrument, *CMAI* Cohen-Mansfield Agitation Inventory, *CSDD* Cornell Scale for Depression in Dementia, *CVF* Category Verbal Fluency, *EPWDS* Engagement of a Person with Dementia Scale, *GDS* Geriatric Depression Scale, *FAB* Frontal Assessment Battery, *HADS* Hospital Anxiety and Depression Scale, *HCS* Holden Communication Scale, *HKSF-DEM* Hong Kong version of Social Functioning in Dementia Scale, *IADL* Instrumental Activities of Daily Living, *IAFAI* Adults and Older Adults Functional Assessment Inventory, *LATA* Language Assessment Test for Aphasia, *MMSE* Mini Mental State Examination, *MOCA-Blind* Montreal cognitive assessment—Blind, *MODA* Milan Overall Dementia Assessment, *NPI* Neuropsychiatric Inventory, *RAID* Rating for Anxiety in Dementia, *QCPR* Quality of the Carer Patient Relationship, *QoL-AD* Quality of Life in Alzheimer's Disease, *RBMT* Rivermead Behavioral Memory Test, *RDRS-2* Rapid Disability Rating Scale-2, *VLMT* Verbal Learning and Memory Test, *WHOQOL-OLD* World Health Organization Quality of Life-OLD, *WMS-R* Wechsler Memory Scale, *ZBI* Zarit Burden Interview

### Effects of digital cognitive stimulation therapy in people with dementia

#### Cognitive function

**Fig. 2:**
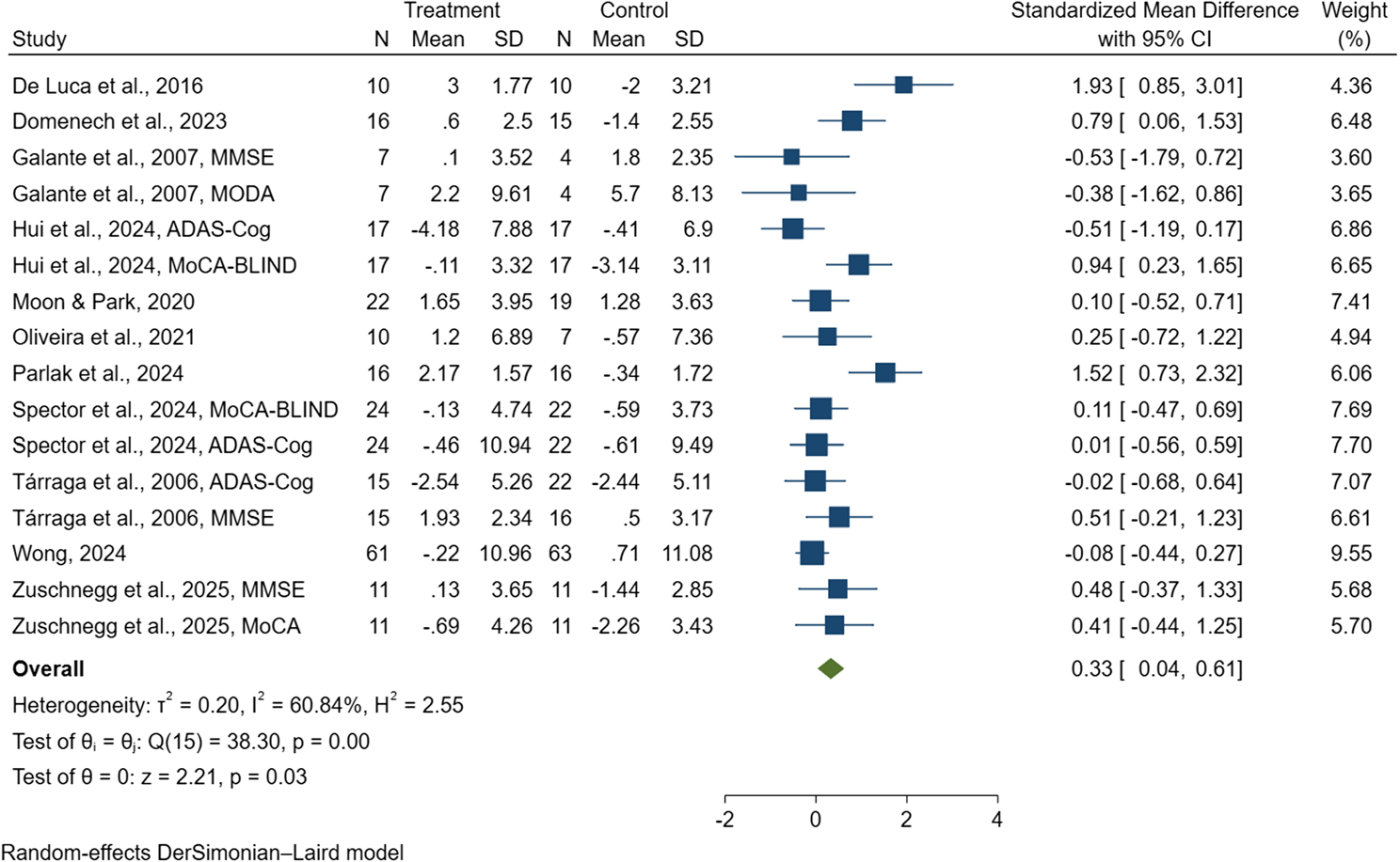
Forest plot of digital cognitive stimulation in people with dementia (Cognitive function)

A total of 11 studies, encompassing 559 people with dementia, were included in the meta-analysis for cognitive function outcomes [38, 47, 81, 82, 85, 88–90, 96, 97, 99]. Due to the variability in cognitive assessment tools used across studies, standardized mean differences (SMDs) were calculated. The cognitive measures included the Mini-Mental State Examination (MMSE) [81, 82, 85, 88–90, 96, 99], the Alzheimer’s Disease Assessment Scale–Cognitive Subscale (ADAS-Cog) [38, 47, 96, 97], the Montreal Cognitive Assessment (MoCA) [99], the Montreal Cognitive Assessment-BLIND (MoCA-BLIND) [38, 47] and Milan Overall Dementia Assessment (MODA) [85]. The pooled SMD was 0.33 (95% CI: 0.04 to 0.62, *p* = 0.03; I^2^ = 61.39%; Q = 38.85, df = 15; see Fig. 2), indicating a statistically significant improvement in cognitive function for participants receiving digital CST compared to those in control conditions such as conventional CST or standard care. The heterogeneity observed was moderate to substantial, suggesting variability across studies, which may be partly attributed to potential publication bias. However, Egger's regression test did not indicate a statistically significant influence of publication bias on the pooled estimate (*p* = 0.427), implying that any such bias was likely minimal.

### Subgroup analyses of cognitive function

#### Cognitive assessment tools

**Fig. 3:**
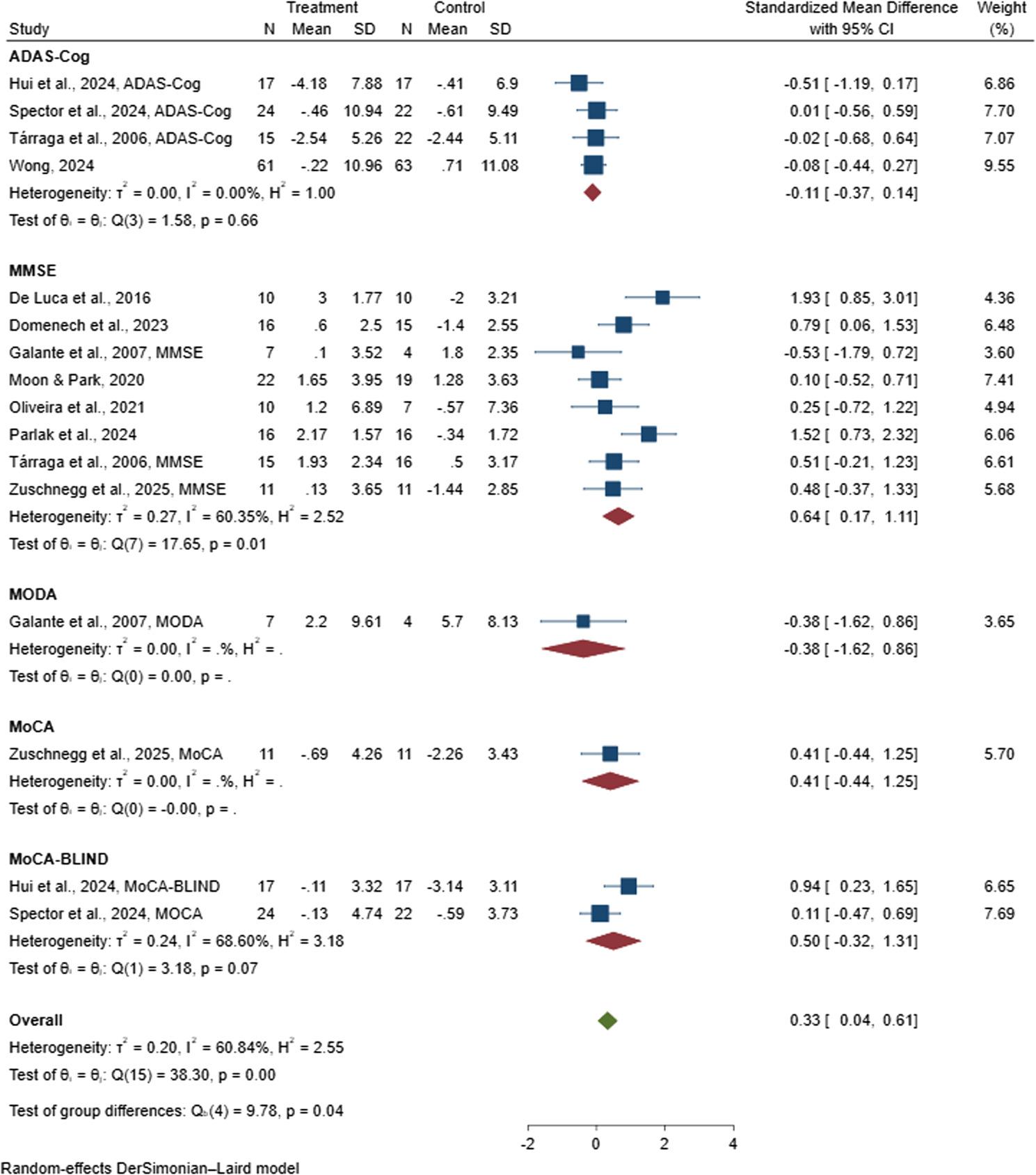
Forest plot of digital cognitive stimulation in people with dementia (Subgroup analyses on cognitive function: Effect assessed based on the cognitive assesment instrument utilized)

Subgroup analysis was conducted based on the type of cognitive assessment instrument used in the 11 included studies, which collectively involved 559 people with dementia and were incorporated into the meta-analysis [38, 47, 81, 82, 85, 88–90, 96, 97, 99]. For studies that employed the ADAS-Cog [38, 47, 96, 97], the pooled SMD was −0.11 (95% CI: −0.37 to 0.14); for MODA [85], the pooled SMD was −0.38 (95% CI: −1.62 to 0.86); for MoCA [99], it was 0.41 (95% CI: −0.44 to 1.25); and for MoCA-BLIND [38, 47], it was 0.50 (95% CI: −0.32 to 1.31), indicating no significant effect of digital CST on cognitive outcomes when assessed using these instruments. In contrast, studies that utilized the MMSE demonstrated a significant positive effect [81, 82, 85, 88–90, 96, 99], with a pooled SMD of 0.64 (95% CI: 0.17 to 1.12), as shown in Fig. 3. These findings suggest that cognitive improvements associated with digital CST were more pronounced when measured using the MMSE compared with the ADAS-Cog, MODA, MoCA, or MoCA-BLIND.

#### Mode of digital delivery

**Fig. 4:**
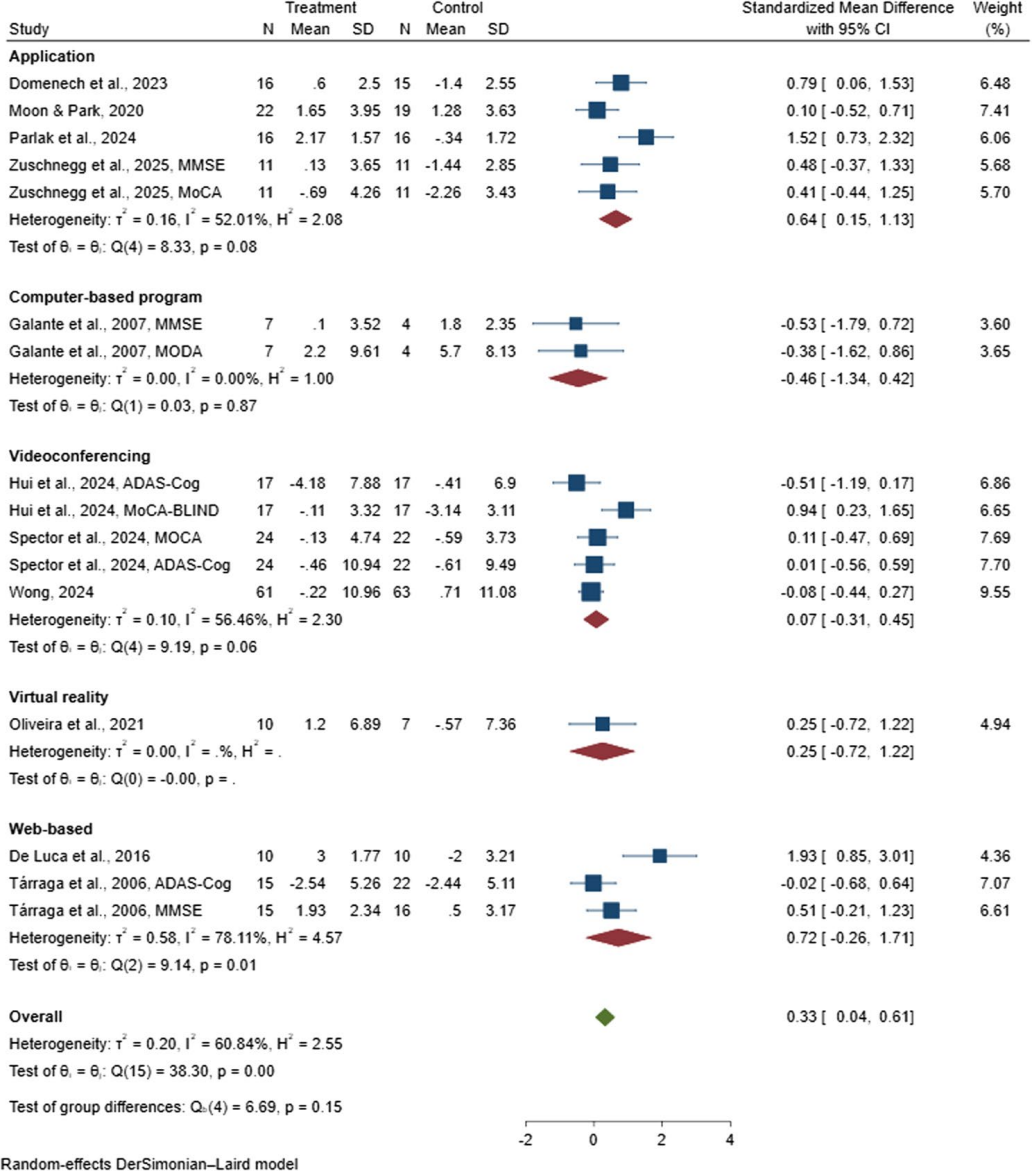
Forest plot of digital cognitive stimulation in people with dementia (Subgroup analyses on cognitive function: Effect assessed by the mode of digital delivery)

Subgroup analysis was conducted to explore the impact of different digital delivery modes on cognitive function in the 11 included studies [38, 47, 81, 82, 85, 88–90, 96, 97, 99], which collectively involved 559 people with dementia and were incorporated into the meta-analysis. The SMD for cognitive outcomes was pooled based on whether the intervention was delivered via mobile applications [82, 88, 90, 99], computer-based program [85], videoconferencing [38, 47, 97], virtual reality [89], and web-based platforms [81, 96]. The pooled SMD for app-based delivery was 0.64 (95% CI: 0.15 to 1.13), suggesting a significant positive effect. In comparison, computer-based programs showed a pooled SMD of −0.46 (95% CI: −1.34 to 0.42), videoconferencing yielded a pooled SMD of 0.07 (95% CI: −0.31 to 0.45), virtual reality-based interventions produced a pooled SMD of 0.25 (95% CI: −0.72 to 1.22), and web-based platforms demonstrated a pooled SMD of 0.73 (95% CI: −0.26 to 1.72), as presented in Fig. 4. These results indicate that CST interventions delivered via mobile applications demonstrated a more consistent and substantial benefit on cognitive function compared with those delivered through computer-based program, videoconferencing, virtual reality or web-based systems.

#### Mode of intervention delivery

**Fig. 5:**
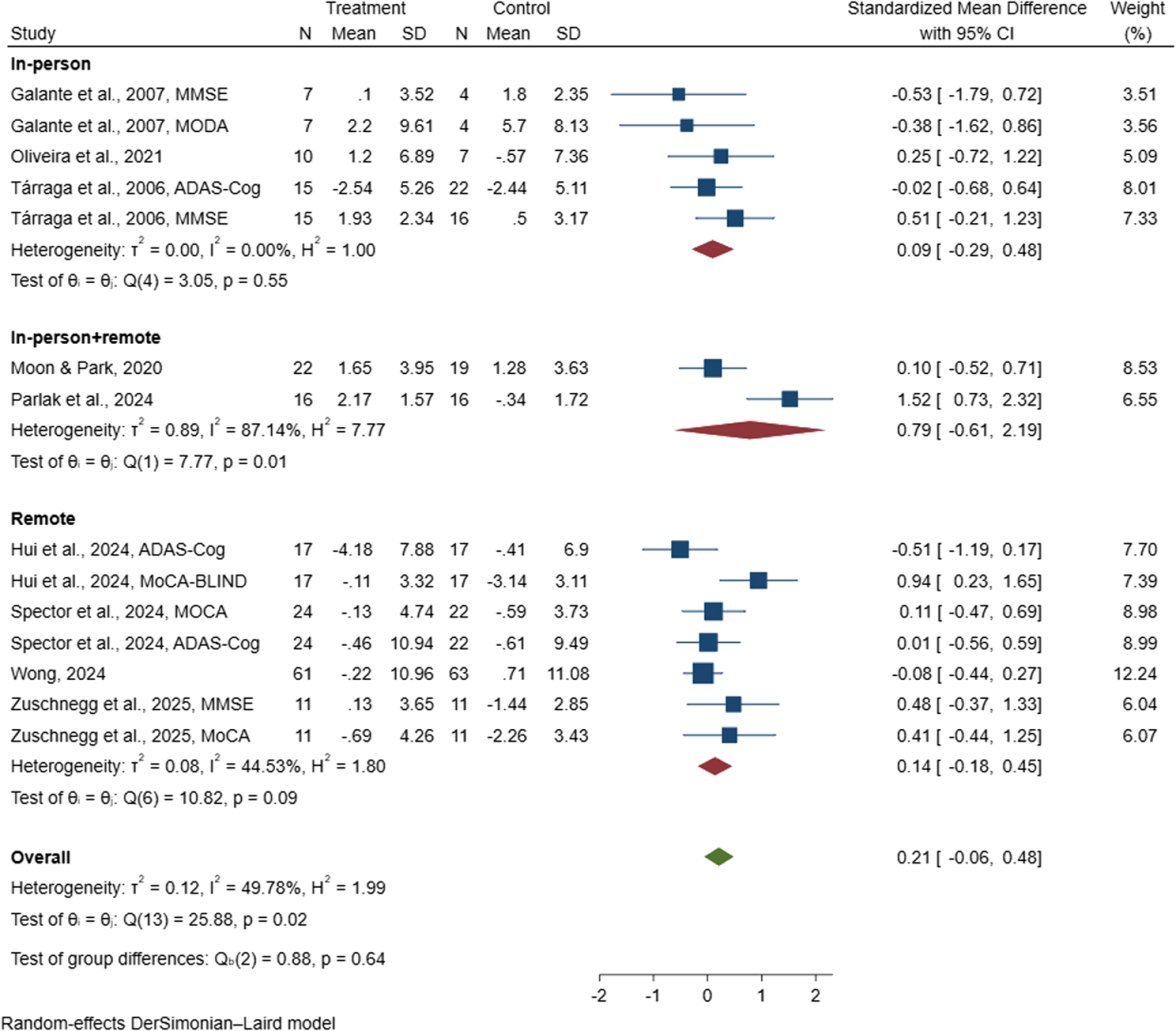
Forest plot of digital cognitive stimulation in people with dementia (Subgroup analyses on cognitive function: Effect assessed by the mode of intervention delivery)

A subgroup analysis was performed to examine the impact of different delivery formats of digital CST on cognitive outcomes in the nine included studies [38, 47, 85, 88–90, 96, 97, 99], which collectively involved 508 people with dementia and were incorporated into the meta-analysis. The pooled SMD for cognitive function were 0.09 (95% CI: −0.29 to 0.48) for in-person delivery [85, 89, 96], 0.79 (95% CI: −0.61 to 2,19) for combined in-person and remote delivery [88, 90], and 0.14 (95% CI: −0.18 to 0.45) for remote-only interventions [38, 47, 97, 99], as illustrated in Fig. 5. Although all modes showed positive trends, none of the differences reached statistical significance. These findings suggest that the mode of digital CST delivery whether conducted in-person, remotely, or through a hybrid format did not significantly influence the overall effectiveness in enhancing cognitive function among people with dementia.

#### Session duration

**Fig. 6:**
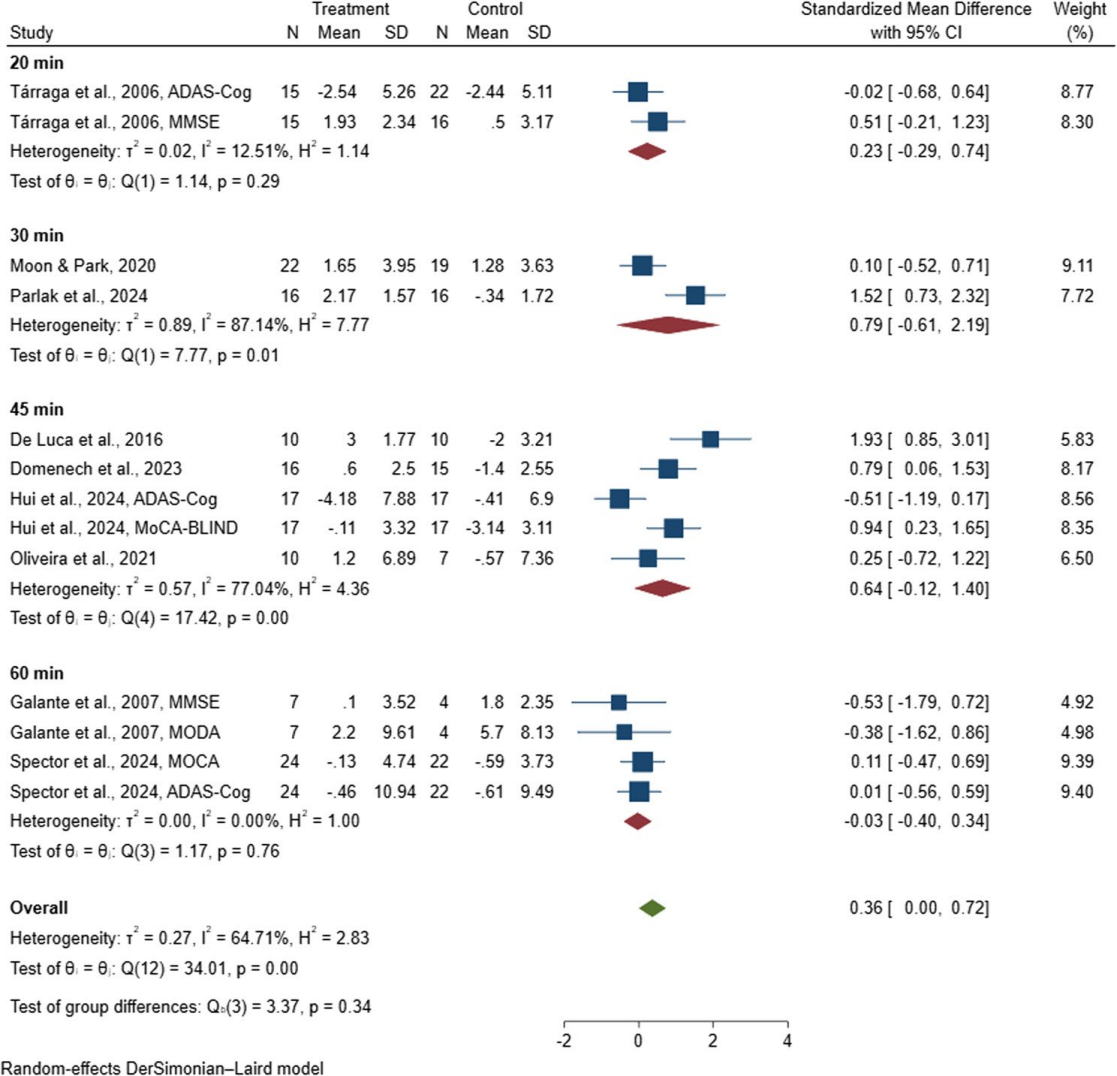
Forest plot of digital cognitive stimulation in people with dementia (Subgroup analyses on cognitive function: Effect assessed by duration of intervention delivered per session)

Subgroup analysis was conducted based on the length of each digital CST session to evaluate its impact on cognitive outcomes in the nine included studies [38, 47, 81, 82, 85, 88–90, 96], which collectively involved 391 people with dementia and were incorporated into the meta-analysis. The pooled standardized mean differences for session durations of 20 [96], 30 [88, 90], 45 [38, 81, 82, 89], and 60 minutes [47, 85] were 0.23 (95% CI: −0.29 to 0.74), 0.79 (95% CI: −0.61 to 2.19), 0.64 (95% CI: −0.12 to 1.40), and −0.03 (95% CI: −0.40 to 0.34), respectively (see Fig. 6). Among these session durations, none demonstrated a statistically significant effect on improving cognitive function in individuals with dementia.

#### Frequency of sessions per week

**Fig. 7:**
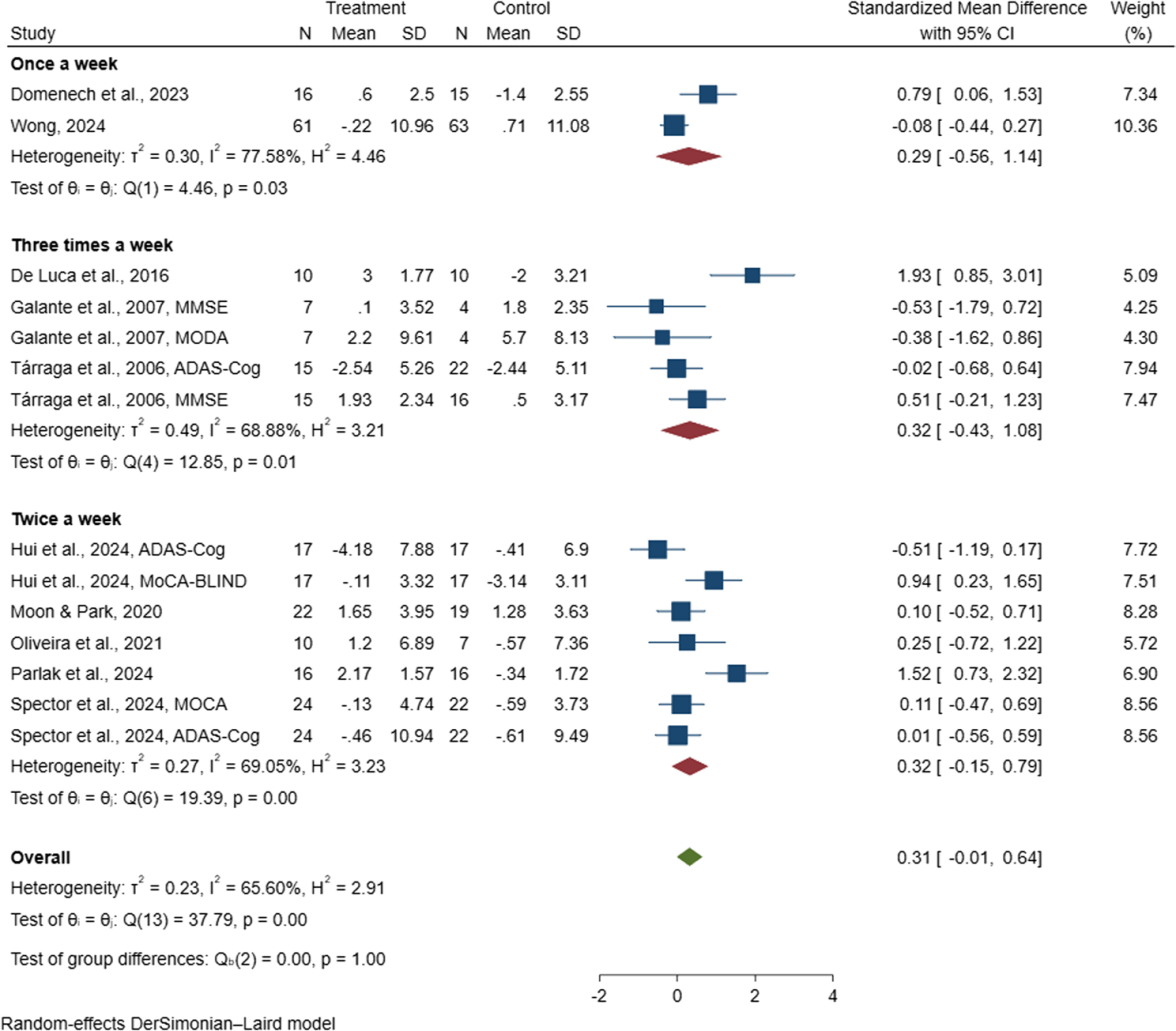
Forest plot of digital cognitive stimulation in people with dementia (Subgroup analyses on cognitive function: Effect assessed based on frequency of sessions per week)

A subgroup analysis was performed to explore how the frequency of digital CST sessions per week influenced cognitive outcomes in the ten included studies [38, 47, 81, 82, 85, 88–90, 96, 97], which collectively involved 515 people with dementia and were incorporated into the meta-analysis. The pooled SMD for interventions delivered once per week was 0.29 (95% CI: −0.56 to 1.14) [82, 97], twice per week was 0.32 (95% CI: −0.15 to 0.79) [38, 47, 88–90], and times per week, the SMD was 0.33 (95% CI: −0.43 to 1.09) [81, 85, 96], as shown in Fig. 7. None of these pooled estimates reached statistical significance, indicating that no specific delivery frequency of digital CST demonstrated a clear benefit for cognitive function. Although the effect sizes for interventions delivered twice or three times per week appeared slightly larger than those delivered once per week, the wide confidence intervals suggest considerable uncertainty. Overall, these findings indicate that the current evidence does not support a definitive optimal weekly frequency for delivering digital CST to improve cognitive outcomes in people with dementia.

#### Total study duration

**Fig. 8:**
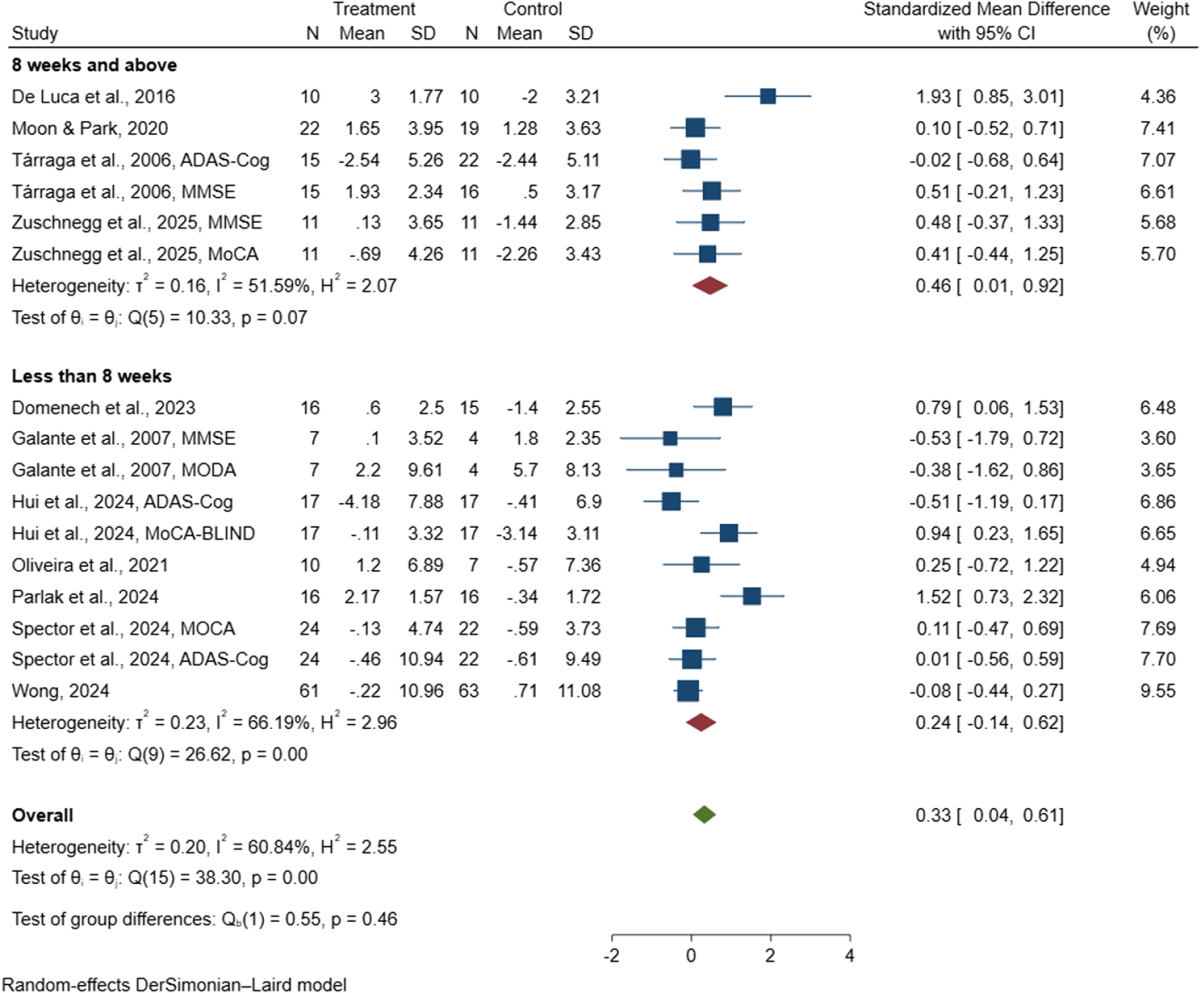
Forest plot of digital cognitive stimulation in people with dementia (Subgroup analyses on cognitive function: Effect assessed based on total duration of the intervention delivered)

A subgroup analysis was conducted to assess the impact of the overall length of the intervention period on cognitive outcomes in the 11 included studies [38, 47, 81, 82, 85, 88–90, 96, 97, 99], which collectively involved 559 people with dementia and were incorporated into the meta-analysis. The pooled SMD for interventions delivered over a period of less than eight weeks was 0.24 (95% CI: −0.14 to 0.63) [38, 47, 82, 85, 89, 90, 97], while interventions lasting eight weeks or more showed a pooled SMD of 0.47 (95% CI: 0.01 to 0.93) [81, 88, 96, 99], as presented in Fig. 8. These findings indicate a statistically significant improvement in cognitive function for digital CST programs implemented for eight weeks or longer. Overall, the results suggest that digital CST programs of at least eight weeks in duration may offer more reliable cognitive benefits for people with dementia.

#### Semantic fluency

**Fig. 9:**
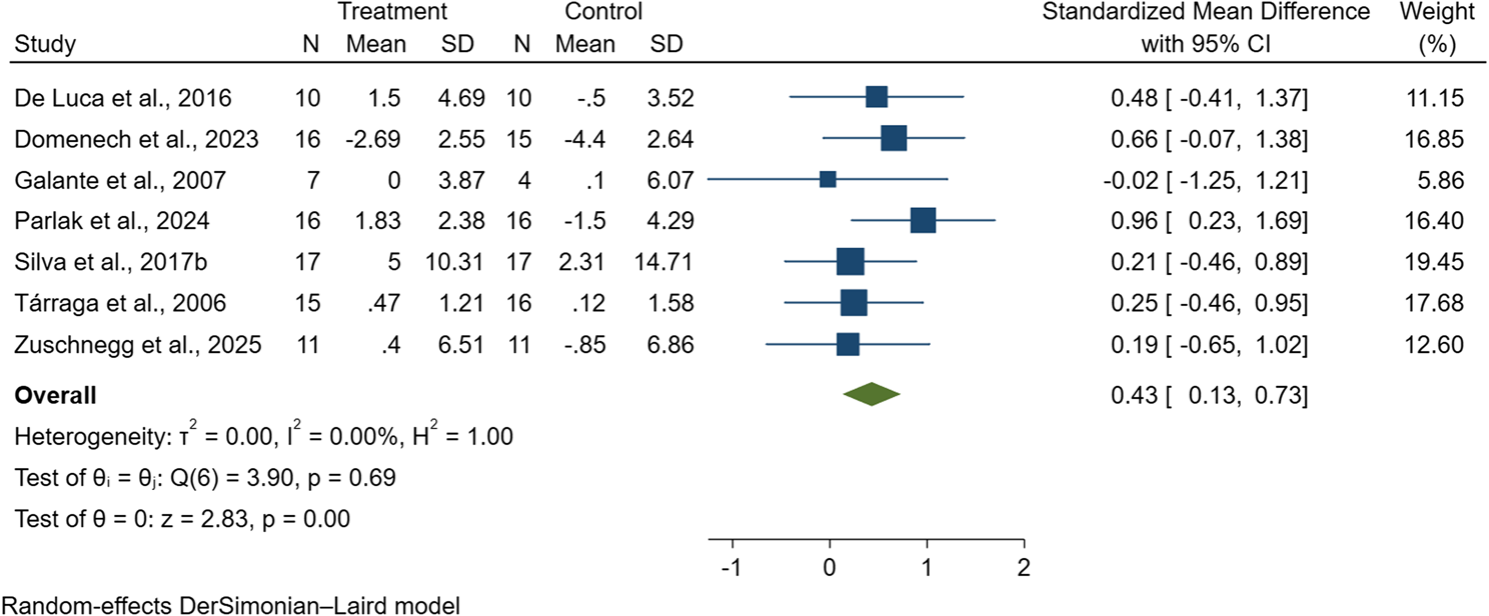
Forest plot of digital cognitive stimulation in people with dementia (Subgroup analyses on cognitive function: Effect assessed based on total duration of the intervention delivered)

A total of 181 participants with dementia from seven studies were included in the meta-analysis assessing semantic fluency outcomes [81, 82, 85, 90, 95, 96, 99]. Due to differences in the assessment instruments used across studies, SMDs were calculated. The tools employed included the Category Verbal Fluency (CVF) [81], the Holden Communication Scale (HCS) [82], the Language Assessment Test for Aphasia (LATA) [90], the Portuguese Letter Fluency Test [95], the Rivermead Behavioral Memory Test (RBMT) [96], and Verbal Learning and Memory Test (VLMT) [99]. The pooled SMD was 0.43 (95% CI: 0.13 to 0.73, *p* < 0.001; I^2^ = 0.00%; Q = 3.91, df = 6; see Fig. 9), indicating a statistically significant improvement in semantic fluency among individuals who received digital CST compared to those receiving traditional CST. The analysis revealed no evidence of heterogeneity. Moreover, Egger’s regression test suggested that publication bias was not a concern (*p* = 0.568), indicating minimal influence of potential bias on the pooled estimate.

#### Depressive symptoms

**Fig. 10:**
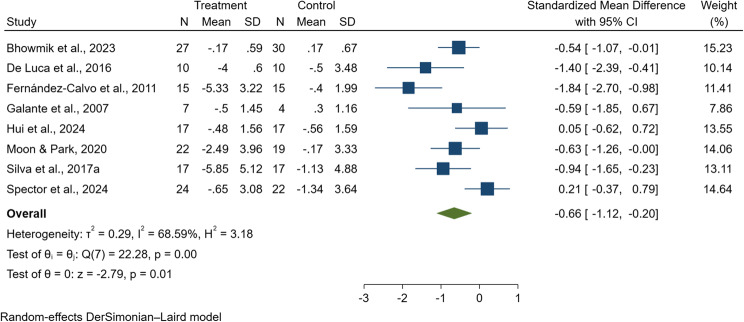
Forest plot of digital cognitive stimulation in people with dementia (Subgroup analyses on cognitive function: Effect assessed based on total duration of the intervention delivered)

A total of 273 people with dementia were included in the meta-analysis from eight studies evaluating depressive outcomes [38, 47, 79, 81, 84, 85, 88, 94]. SMDs were computed due to the use of different depression assessment tools across studies. These included the Cornell Scale for Depression in Dementia (CSDD) [84, 88], the Geriatric Depression Scale (GDS) [47, 81, 85, 94], the Geriatric Depression Scale-15 items (GDS-15) [38], the Neuropsychiatric Inventory (NPI) [79]. The pooled SMD was −0.66 (95% CI: −1.12 to −0.20, *p* = 0.01; I^2^ = 68.94%; Q = 22.54, df = 7; see Fig. 10), indicating a significant reduction in depressive symptoms among participants who received digital CST compared to those receiving traditional CST. The heterogeneity observed was moderate to substantial, suggesting variability across studies, which may be partly attributed to potential publication bias. However, Egger’s regression test revealed no significant indication of publication bias (*p* = 0.207), supporting the reliability of the pooled effect estimate.

#### Quality of life

**Fig. 11:**
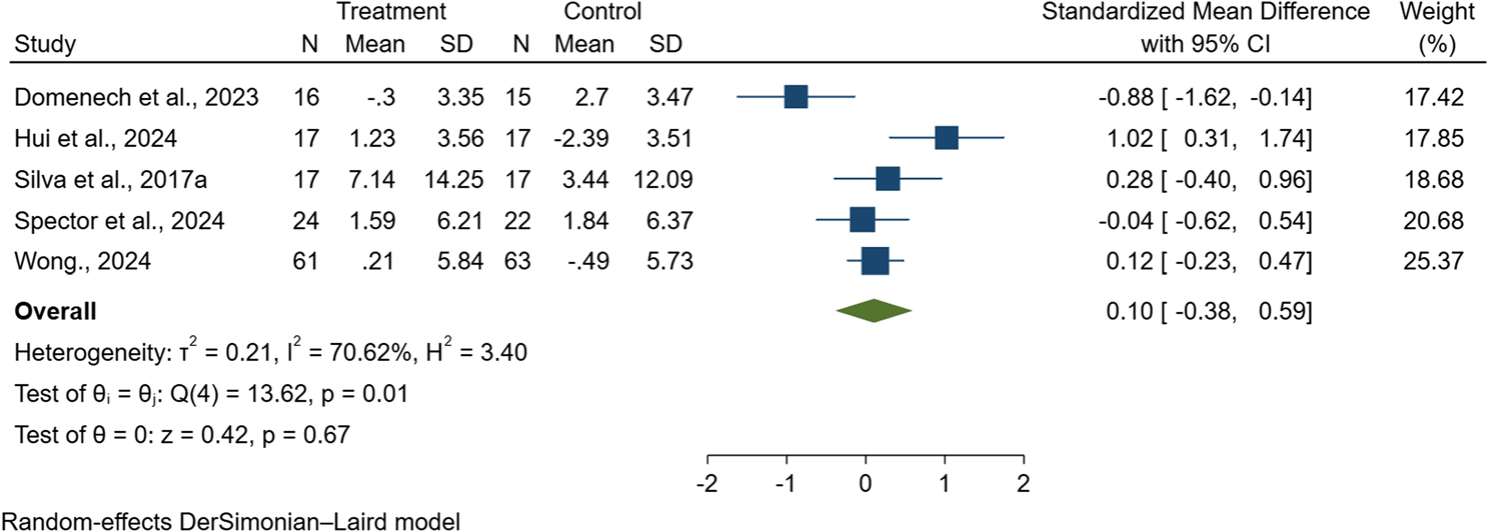
Forest plot of digital cognitive stimulation in people with dementia (Subgroup analyses on cognitive function: Effect assessed based on total duration of the intervention delivered)

Five studies, encompassing a total of 269 people with dementia, were included in the meta-analysis assessing quality of life outcomes [38, 47, 82, 94, 97]. Due to variation in the instruments used to evaluate this domain, standardized mean differences were calculated. The quality of life measures employed included the Quality of Life in Alzheimer's Disease (QoL-AD) scale [38, 47, 82, 97] and World Health Organization Quality of Life-OLD (WHOQoL-OLD) [94]. The pooled SMD was 0.10 (95% CI: −0.38 to 0.59; *p* = 0.67; I^2^ = 70.81%; Q = 13.70, df = 4; see Fig. 11), indicating no statistically significant difference in post-intervention quality of life between individuals receiving digital CST and those in traditional CST. Moderate heterogeneity was observed across the studies. Egger’s regression test revealed no evidence of publication bias (*p* = 0.955), suggesting minimal influence on the overall estimate.

### Sensitivity analysis

A sensitivity analysis was conducted using the leave-one-out approach, where the study contributing the greatest weight to each pooled analysis was sequentially removed to test the robustness of the findings. The results remained non-significant in all outcomes following the exclusion of the most influential study, suggesting that the overall estimates were stable. Specifically, *p*-values were as follows: cognitive function (*p* = 0.62), semantic fluency (*p* = 0.72), depressive symptoms (*p* = 0.62), and quality of life (*p* = 0.88). These findings indicate that no single study unduly influenced the pooled effect sizes.

#### Publication bias and GRADE evidence

The methodological quality of the included studies was evaluated using the RoB-2 tool for randomized controlled trials and the ROBINS-I tool for non-randomized studies. While no studies were excluded based on high risk of bias, several domains exhibited potential sources of bias. Common issues identified included lack of blinding among participants, assessors, and interventionists, dropout rates exceeding 20%, and the absence of pre-registered study protocols.

The certainty of evidence was further assessed using the GRADEpro GDT framework. Overall, the quality of evidence ranged from low to moderate. Downgrading for inconsistency was applied due to substantial heterogeneity (I^2^ > 40%), while imprecision was downgraded because the total sample size was below 400 and the confidence intervals for the standardized mean differences (SMDs) overlapped between intervention and control groups. A detailed summary of the evidence grading is presented in Table 2.

**Table 2:** GRADE Evidence Digital cognitive stimulation therapy compared to usual care for people with dementia Bibliography

**Certainty assessment**							**Summary of findings**				
**Participants (studies) Follow-up**	**Risk of bias**	**Inconsistency**	**Indirectness**	**Imprecision**	**Publication bias**	**Overall certainty of evidence**	**Study event rates (%)**		**Relative effect (95% CI)**	**Anticipated absolute effects**	
							**With usual care**	**With Digital cognitive stimulation therapy**		**Risk with usual care**	**Risk difference with Digital cognitive stimulation therapy**
Cognitive function											
559 (11 RCTs)	not serious	serious^a^	not serious	serious	none	⨁⨁⨁◯ Moderate^a^	276	283	-	-	SMD 0.33 SD higher (0.04 higher to 0.61 higher)
Semantic fluency											
181 (7 RCTs)	not serious	serious	not serious	serious^b^	none	⨁⨁⨁◯ Moderate^b^	89	92	-	-	SMD 0.43 SD higher (0.13 higher to 0.73 higher)
Depressive symptom											
273 (8 RCTs)	not serious	serious^a^	not serious	serious^b^	none	⨁⨁◯◯Low^a,b^	238	251	-	-	SMD −0.66 SD higher (−1.12 higher to −0.20 higher)
Quality of life											
269 (5 RCTs)	not serious	serious^a^	not serious	serious^b,c^	none	⨁⨁◯◯Low^a,b,c^	134	135	-	-	SMD 0.10 SD higher (−0.38 higher to 0.59 higher)

*CI* Confidence interval, *SMD* Standardised mean difference

^a^Consistency was downgraded because the heterogeneity was superior to 40%

^b^Precision was downgraded because the number of observations was inferior to 400

^c^Precision was downgraded because the SMD confidence interval overlapped between experimental and control intervention

## Discussion

This systematic review and meta-analysis incorporated data from 18 studies encompassing 756 individuals diagnosed with dementia. The objective was to assess the impact of digital CST on cognitive performance, semantic fluency, depressive symptoms, and quality of life. The analysis revealed that digital CST led to significant enhancements in cognitive function and semantic fluency, as well as a reduction in depressive symptoms, when compared to traditional CST modalities. Subgroup analyses identified that interventions utilizing digital CST, employing the MMSE as an assessment tool, and administered biweekly in 45-minute sessions over a duration of less than eight weeks were particularly effective in improving cognitive outcomes. However, no substantial differences were observed in quality of life between participants receiving digital CST and those undergoing traditional CST. The absence of a significant difference in quality-of-life outcomes between digital and traditional CST suggested that digital CST can deliver comparable benefits to conventional approaches. This finding highlighted the potential of digital CST as a scalable and resource-efficient alternative, particularly valuable in settings with limited access to in-person interventions. This meta-analysis was the first to quantitatively evaluate the effectiveness of CST for people with dementia. A previous systematic review on technology-assisted reminiscence therapy, which included 44 studies, reported several benefits, including enhanced access to rich and engaging multimedia content, increased involvement in social interactions, and reduced motor challenges during media engagement [56]. However, the review was constrained by limited descriptions of the therapeutic methods used and a lack of detailed analysis regarding how outcomes varied according to subtypes of types of dementia or intervention characteristics. Another published review investigating computer-based cognitive interventions for people with dementia demonstrated encouraging outcomes in improving cognitive performance and in reducing symptoms of anxiety and depression [55]. Despite these positive results, the review incorporated a wide range of cognitive approaches, such as cognitive stimulation and cognitive training, into aggregated analyses. The relatively small sample sizes in each subgroup may have introduced bias and limited the generalizability of the findings (fewer than 10 studies). Given the multifaceted needs of people with dementia, including the preservation of cognitive function, the promotion of psychological well-being, and the enhancement of overall quality of life, focused research on specific intervention modalities is essential. Investigating targeted approaches such as cognitive stimulation therapy can lead to a more precise and comprehensive understanding of both the mechanisms of action and the broader therapeutic outcomes across cognitive, emotional support, and functional domains.

### Comparison between digital and traditional CST delivery

The findings of this review reveal that, compared to traditional CST, digital CST led to greater improvements in cognitive function. This result is consistent with previous trials, which also reported significant cognitive benefits following digital CST in people with dementia [81, 82, 89, 90, 96]. Digital CST delivered through web-based platforms, mobile applications, computer programs, or virtual reality systems appears to be a promising, practical, and effective tool for cognitive stimulation. Incorporating digital technology into CST delivery may offer additional advantages, such as greater accessibility and more engaging content, which can improve adherence to therapy and contribute to cognitive enhancement [100]. Moreover, digital CST supported personalized training, allowing activities to be tailored to individual preferences. This personalization provides a more immersive, enjoyable, and flexible experience [101]. Such adaptability can help maintain user interest and make digital CST a viable option for both short-term and long-term use in preserving cognitive function, with minimal risk for people with dementia [102].

Subgroup analyses in this review should be interpreted with caution because the number of studies and participants within each subgroup was small, limiting statistical precision. In addition, differences in delivery modality often coincided with differences in outcome measures (e.g., use of the MMSE), making it difficult to disentangle the effect of the intervention from the assessment tool. Therefore, any apparent differences should be considered exploratory rather than evidence of superiority. Subgroup analyses revealed that digital CST interventions delivered for eight weeks or longer tended to show more favorable trends in cognitive outcomes compared with shorter protocols. This pattern aligns with evidence suggesting that extended cognitive stimulation is necessary to support neuroplastic adaptation and meaningful cognitive reinforcement in people with dementia [37, 103]. Longer intervention periods may enable repeated practice, consolidation of cognitive strategies, and more sustained engagement, which are essential for achieving detectable improvements in global cognition. However, despite these observable trends, none of the pooled subgroup estimates reached statistical significance, indicating that no specific delivery frequency, session duration, or modality (in-person, remote, or digital) demonstrated a reliably superior benefit for cognitive function. These findings echo recent meta-analytic work showing that while CST can improve cognition and quality of life, effect sizes vary widely and are influenced by methodological heterogeneity and implementation factors [24, 104]. Consequently, although longer intervention periods may be theoretically advantageous and have shown directionally positive effects, the current evidence does not support a definitive optimal dosing schedule for digital CST.

The findings of this study suggested that CST delivered via mobile applications has shown superior outcomes in improving cognitive function. This advantage may stem from the individualized nature of app-based CST, which allows for tailored, engaging experiences that promote sustained user interaction. Previous research has demonstrated that app-based CST enhances user engagement by fostering deeper cognitive interaction, which in turn has been linked to improved concentration and memory retention [105]. In contrast, digital platforms such as computer-based programs, videoconferencing, virtual reality (VR), and web-based applications face inherent limitations that hinder their effectiveness in delivering CST to older adults with dementia. Many older patients encounter technical and usability barriers, including low digital literacy, lack of device or internet access, and the need for caregiver assistance – which limit adoption and often lead to the exclusion of those with sensory impairments [106, 107]. Additionally, engaging with digital interfaces can impose a higher cognitive load and increase the risk of overstimulation; for example, complex menus or visually busy designs may overwhelm users’ cognitive capacity [108], while immersive VR interventions have been reported to cause disorientation, nausea, and other sensory side effects in this population [109]. Moreover, the quality of therapeutic engagement is often diminished in these digital delivery formats, as remote programs typically offer less personalization and weaker social interaction. Many digital CST applications provide only static, one-size-fits-all activities with minimal tailoring to individual needs [108], and participants frequently struggle to establish the same interpersonal connection and interactive engagement through online sessions as they do during in-person CST [110].

Finally, cognitive outcomes measured using the MMSE showed greater improvements than those assessed with the ADAS-Cog, MODA, MoCA, MoCA-BLIND. This discrepancy may be due to the differing sensitivities of these instruments in detecting cognitive changes at different stages of dementia [111]. The MMSE, although widely used, is a brief global screening tool and is known to have limited sensitivity for detecting subtle or domain-specific cognitive changes, particularly in individuals with mild or early-stage dementia [112, 113]. In contrast, the ADAS-Cog provides more extensive assessment across several domains, including memory, language, and praxis; however, its responsiveness in mild dementia is often reduced due to floor effects and limited dynamic range [114, 115]. Similarly, the MoCA and its adapted versions, while more sensitive than the MMSE for detecting mild cognitive impairment, tend to capture smaller incremental changes that may not translate into large, standardized effect sizes in intervention studies [116, 117].

### Semantic fluency

The pooled analysis demonstrated that digital CST produced significantly greater improvements in semantic fluency among people with dementia compared to traditional CST approaches. This improvement was likely due to the multimodal stimulation provided by integrated audiovisual components, which simultaneously engaged visual and auditory neural pathways to enhance both encoding and retrieval processes [15, 118]. Additionally, these digital platforms employed adaptive algorithms that adjusted task difficulty in real time based on each user’s performance, maintaining an optimal challenge level to support neuroplasticity in language and executive functions [119, 120]. Finally, immediate feedback and opportunities for repeated practice helped consolidate semantic memories and further improved verbal fluency in individuals receiving digital CST.

### Depressive symptom

The meta-analysis showed that, compared with traditional CST, adding digital CST led to a reduction in depressive symptoms in people with dementia [81, 84, 88, 95]. The application of different CST formats, including face-to-face and paper-based activities [24], game-based cognitive tasks [98, 105], and personalized memory or reminiscence exercises that involve recalling past events, names, or sequences [82, 85], appears to promote greater engagement and enjoyment when adapted for digital delivery. These approaches have also been linked to improvements in mood and psychological well-being in people with dementia [47, 103, 121]. Engaging in activities that were both enjoyable and mentally stimulating likely triggered the release of β-endorphins chemicals known to lower stress hormones, ease pain, and promote feelings of well-being [122]. These changes at both the chemical and emotional connectivity levels likely explained the reduction in depressive symptoms. To better understand how digital CST functioned and to refine treatment guidelines, additional randomized controlled trials were needed to explore which elements and dosages were most effective for people with dementia.

### No changes in quality of life

Meta-analysis did not demonstrate a significant effect of CST on quality of life in people with dementia. One possible explanation for these non-significant findings was the limited number of studies included in the pooled analysis (*n* = 3), which may have lacked sufficient statistical power to detect a meaningful intervention effect. Additionally, variability in the mode of digital delivery and differences in intervention characteristics, such as the duration and intensity of the CST, might have contributed to heterogeneity, thereby affecting the overall outcomes and potentially introducing bias. Furthermore, variations in the severity of dementia among participants across studies might have influenced the observed effectiveness of digital CST.

## Implication for practice

The results of this systematic review and meta-analysis underscore the practical value of implementing digital CST as an effective intervention for people with dementia. When administered in 45-minute sessions twice weekly over a short-term duration of fewer than eight weeks, digital CST was associated with measurable improvements in cognitive function, semantic fluency, and symptoms of depression. The adaptable and interactive features of digital CST platforms supported greater patient engagement and adherence, presenting a scalable alternative to conventional paper-based formats.

Moreover, healthcare providers should consider adopting digital CST as part of a multidisciplinary approach to dementia care, particularly in settings aiming to expand access to non-pharmacological therapies. Digital CST may be especially beneficial for individuals who cannot attend face-to-face sessions due to mobility challenges, geographic isolation, lack of trained facilitators, or a preference for flexible, self-paced engagement. Incorporating digital CST into care routines may enhance cognitive and related outcomes while offering a cost-effective and patient-centered tool to complement existing interventions. Platforms should be continuously refined to match the needs of people with dementia, with real-time feedback and appropriate content complexity. Furthermore, actively involving care partners in the implementation process may mitigate barriers related to technology adoption [123]. Their involvement can support navigation, troubleshoot technological difficulties, and mitigate anxiety or resistance commonly associated with digital interventions. Training modules for care partners and healthcare providers may further enhance uptake and ensure fidelity of delivery. To maximize equitable implementation, it is also crucial to address socioeconomic barriers, such as high costs, limited internet access, and low digital literacy among lower-income groups, which may otherwise restrict access and widen disparities. Strategies like subsidized device provision, accessible platform design, and integration into public health services can support broader adoption across diverse populations.

### Limitations

This meta-analysis had several limitations that warrant consideration. Despite conducting an extensive search across multiple databases, grey literature, and manual screening, it is possible that some relevant studies were inadvertently excluded. Some studies did not provide enough data to calculate SMDs, and efforts to obtain the missing information from corresponding authors were unsuccessful. Subgroup analyses were limited to cognitive function outcomes. Expanding these analyses to other domains could have provided more comprehensive insights. Furthermore, the inability to perform subgroup analyses based on dementia type and dementia severity may have influenced the observed effects of digital CST. In addition, the small number of studies and participants available for subgroup analyses reduced statistical power and precluded formal comparisons between digital delivery modalities. Subgroup findings were vulnerable to confounding by correlated study characteristics, such as outcome measurement tools, and should therefore be interpreted as exploratory rather than evidence of comparative efficacy or superiority. Additionally, despite the promising outcomes, the existing evidence base is geographically narrow, with studies concentrated in high-income countries such as Australia, Italy, Spain, the United Kingdom, and Japan. There is a critical gap in data from low- and middle-income countries (LMICs), as well as from technologically advanced yet underrepresented regions, including South Korea. This limits the external validity and cross-cultural applicability of current findings. To address this, future investigations should prioritize culturally sensitive adaptation and evaluation of digital CST interventions across diverse socioeconomic and cultural-linguistic contexts. 

## Conclusion

Our analysis suggests that digital CST for people with dementia effectively improves cognitive function, semantic fluency, and depressive symptoms. Subgroup analyses indicated that delivering the intervention twice a week for 45 minutes over a period of fewer than eight weeks yielded the most significant cognitive improvements. These findings support the potential of digital technology as a viable and acceptable modality for implementing dementia care. Digital CST appears well-suited to address the needs of people with dementia by offering a variety of enjoyable, cognitively stimulating activities that target thinking, memory, and language. The use of digital platforms enhances engagement, personalization, and accessibility for both participants and caregivers. While the study showed promising results for cognitive and emotional connectivity outcomes, no significant improvements were observed in quality of life. This may be attributed to the high variability in study designs and the limited number of studies included in the pooled analysis. Additionally, the severity of dementia likely influences the effectiveness of the intervention; however, we were unable to perform stratified analyses based on dementia severity due to the small number of studies reporting this variable and the uneven distribution of severity levels. Future research should aim to explore the differential effects of digital CST across varying stages of dementia and further evaluate its impact on a broader range of outcomes.

## Supplementary Information

Supplementary Material 1.

## Data Availability

Data sharing is not applicable to this article as no new data were created or analyzed in this study.
