# Supplementary material for: Efficacy of digital cognitive stimulation therapy for people with dementia: a systematic review and meta-analysis
Source: BMC Geriatr. 2026 Apr 6;26:700. doi: 10.1186/s12877-026-07180-9 (PMC13191879; doi:10.1186/s12877-026-07180-9)
Supplement: Supplementary file 1 — Supplementary Material 1. [file 12877_2026_7180_MOESM1_ESM.docx]

**Appendix A**
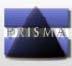


**PRISMA 2020 Checklist**

| **Section and Topic** | **Item #** | **Checklist item** | **Location where item is reported (Page)** |  |  |
| --- | --- | --- | --- | --- | --- |
| **TITLE** | | |  |  |  |
| Title | 1 | Identify the report as a systematic review. | 1 |  |  |
| **ABSTRACT** | | |  |  |  |
| Abstract | 2 | See the PRISMA 2020 for Abstracts checklist. | 1 |  |  |
| **INTRODUCTION** | | |  |  |  |
| Rationale | 3 | Describe the rationale for the review in the context of existing knowledge. | 2-4 |  |  |
| Objectives | 4 | Provide an explicit statement of the objective(s) or question(s) the review addresses. | 4 |  |  |
| **METHODS** | | |  |  |  |
| Eligibility criteria | 5 | Specify the inclusion and exclusion criteria for the review and how studies were grouped for the syntheses. | 5-6 |  |  |
| Information sources | 6 | Specify all databases, registers, websites, organisations, reference lists and other sources searched or consulted to identify studies. Specify the date when each source was last searched or consulted. | 5 |  |  |
| Search strategy | 7 | Present the full search strategies for all databases, registers and websites, including any filters and limits used. | Appendix B |  |  |
| Selection process | 8 | Specify the methods used to decide whether a study met the inclusion criteria of the review, including how many reviewers screened each record and each report retrieved, whether they worked independently, and if applicable, details of automation tools used in the process. | 6 |  |  |
| Data collection process | 9 | Specify the methods used to collect data from reports, including how many reviewers collected data from each report, whether they worked independently, any processes for obtaining or confirming data from study investigators, and if applicable, details of automation tools used in the process. | 6 |  |  |
| Data items | 10a | List and define all outcomes for which data were sought. Specify whether all results that were compatible with each outcome domain in each study were sought (e.g. for all measures, time points, analyses), and if not, the methods used to decide which results to collect. | 6 |  |  |
|  | 10b | List and define all other variables for which data were sought (e.g. participant and intervention characteristics, funding sources). Describe any assumptions made about any missing or unclear information. | 6 |  |  |
| Study risk of bias assessment | 11 | Specify the methods used to assess risk of bias in the included studies, including details of the tool(s) used, how many reviewers assessed each study and whether they worked independently, and if applicable, details of automation tools used in the process. | 6-7 |  |  |
| Effect measures | 12 | Specify for each outcome the effect measure(s) (e.g. risk ratio, mean difference) used in the synthesis or presentation of results. | 7-8 |  |  |
| Synthesis methods | 13a | Describe the processes used to decide which studies were eligible for each synthesis (e.g. tabulating the study intervention characteristics and comparing against the planned groups for each synthesis (item #5)). | 7-8 |  |  |
|  | 13b | Describe any methods required to prepare the data for presentation or synthesis, such as handling of missing summary statistics, or data conversions. | 7-8 |  |  |
|  | 13c | Describe any methods used to tabulate or visually display results of individual studies and syntheses. | 7-8 |  |  |
|  | 13d | Describe any methods used to synthesize results and provide a rationale for the choice(s). If meta-analysis was performed, describe the model(s), method(s) to identify the presence and extent of statistical heterogeneity, and software package(s) used. | 7-8 |  |  |
|  | 13e | Describe any methods used to explore possible causes of heterogeneity among study results (e.g. subgroup analysis, meta-regression). | 8 |  |  |
|  | 13f | Describe any sensitivity analyses conducted to assess robustness of the synthesized results. | 8 |  |  |
| Reporting bias assessment | 14 | Describe any methods used to assess risk of bias due to missing results in a synthesis (arising from reporting biases). | 6-7 |  |  |
| Certainty assessment | 15 | Describe any methods used to assess certainty (or confidence) in the body of evidence for an outcome. | 7 |  |  |
| **RESULTS** | | |  |  |  |
| Study selection | 16a | Describe the results of the search and selection process, from the number of records identified in the search to the number of studies included in the review, ideally using a flow diagram. | 8, Figure 1 |  |  |
|  | 16b | Cite studies that might appear to meet the inclusion criteria, but which were excluded, and explain why they were excluded. | 8-9 |  |  |
| Study characteristics | 17 | Cite each included study and present its characteristics. | 9-10, Table 1 |  |  |
| Risk of bias in studies | 18 | Present assessments of risk of bias for each included study. | 14 |  |  |
| Results of individual studies | 19 | For all outcomes, present, for each study: (a) summary statistics for each group (where appropriate) and (b) an effect estimate and its precision (e.g. confidence/credible interval), ideally using structured tables or plots. | Figure 2 |  |  |
| Results of syntheses | 20a | For each synthesis, briefly summarise the characteristics and risk of bias among contributing studies. | 14 |  |  |
|  | 20b | Present results of all statistical syntheses conducted. If meta-analysis was done, present for each the summary estimate and its precision (e.g. confidence/credible interval) and measures of statistical heterogeneity. If comparing groups, describe the direction of the effect. | 10-14 |  |  |
|  | 20c | Present results of all investigations of possible causes of heterogeneity among study results. | 10-14 |  |  |
|  | 20d | Present results of all sensitivity analyses conducted to assess the robustness of the synthesized results. | 10-14 |  |  |
| Reporting biases | 21 | Present assessments of risk of bias due to missing results (arising from reporting biases) for each synthesis assessed. | Appendix C |  |  |
| Certainty of evidence | 22 | Present assessments of certainty (or confidence) in the body of evidence for each outcome assessed. | 14 |  |  |
| **DISCUSSION** | | |  |  |  |
| Discussion | 23a | Provide a general interpretation of the results in the context of other evidence. | 14-20 |  |  |
|  | 23b | Discuss any limitations of the evidence included in the review. | 20-21 |  |  |
|  | 23c | Discuss any limitations of the review processes used. | 20-21 |  |  |
|  | 23d | Discuss implications of the results for practice, policy, and future research. | 19-20 |  |  |
| **OTHER INFORMATION** | | |  |  |  |
| Registration and protocol | 24a | Provide registration information for the review, including register name and registration number, or state that the review was not registered. | 4 |  |  |
|  | 24b | Indicate where the review protocol can be accessed, or state that a protocol was not prepared. | 4 |  |  |
|  | 24c | Describe and explain any amendments to information provided at registration or in the protocol. | 4 |  |  |
| Support | 25 | Describe sources of financial or non-financial support for the review, and the role of the funders or sponsors in the review. | 21 |  |  |
| Competing interests | 26 | Declare any competing interests of review authors. | 21 |  |  |
| Availability of data, code and other materials | 27 | Report which of the following are publicly available and where they can be found: template data collection forms; data extracted from included studies; data used for all analyses; analytic code; any other materials used in the review. | 22 |  |  |

*From:*  Page MJ, McKenzie JE, Bossuyt PM, Boutron I, Hoffmann TC, Mulrow CD, et al. The PRISMA 2020 statement: an updated guideline for reporting systematic reviews. BMJ 2021;372:n71. doi: 10.1136/bmj.n71

**Appendix B**

**PICO Worksheet and Search Strategy Protocol**

PICO of digital cognitive stimulation therapy for people with dementia

| **Components** | **Items** | **Keywords** |
| --- | --- | --- |
| **Population** | People with dementia | Dementia, Alzheimer's disease, Lewy body, parkinson’s dementia, frontotemporal dementia, mixed dementia, vascular dementia |
| **Intervention** | Cognitive stimulation therapy | Cognitive stimulation therapy, CST, individual cognitive stimulation therapy, iCST, maintenance cognitive stimulation therapy, mCST, cognitive intervention, cognitive stimulation, memory stimulation, memory therapy, reality orientation, memory groups, memory support, global stimulation, cognitive psychostimulation |
|  | Digital health | Digital health, digital, digital medicine, digital intervention, digital health care, electronic health, ehealth, mobile heatlh, mhealth, technology, apps, applications, mobile app, wearable devices, virtual reality, augmented reality, computer platform, web-based, videoconferencing, video conference, virtual meeting, Zoom, Google Meet |
| **Comparison** | Usual care | Usual care, treatment as usual, conventional CST, or Any CST activities that do not involve delivery with technology |
| **Study design** | Experimental study | Randomized controlled trial, rct, randomized control trial, controlled trial, randomised controlled trial, randomization, quasi-experimental, pre-post test design, experimental study, interventional study |

| Search strategies of each database | | |
| --- | --- | --- |
| Database | Index and keyword terms | Result |
| CINAHL Plus with Full text | XB (Dementia) OR XB (alzheimer's disease) OR XB (lewy body dementia) OR XB (parkinson's dementia) OR XB (frontotemporal dementia) OR XB (mixed dementia) OR XB (vascular dementia) AND XB (cognitive stimulation therapy) OR XB (cst) OR XB (individual cognitive stimulation therapy) OR XB (iCST) OR XB (maintenance cognitive stimulation therapy) OR XB (mCST) OR XB (cognitive intervention) OR XB (memory group) OR XB (reality orientation) OR XB (memory support) OR XB (global stimulation) OR XB (cognitive psychostimulation) AND XB (digital health) OR XB (digital) OR XB (digital medicine) OR XB (digital intervention) OR XB (digital health care) OR XB (electronic health) OR XB (ehealth) OR XB (mobile health) OR XB (mhealth) OR XB (technology) OR XB (apps) OR XB (applications) OR XB (mobile app) OR XB (wearable devices) OR XB (virtual reality) OR XB (augmented reality) OR XB (computer platform) OR XB (web-based) OR XB (videoconferencing) OR XB (video conference) OR XB (virtual meeting) OR XB (zoom) OR XB (google meet) AND XB (randomized controlled trials) OR XB (rct) OR XB (randomized control trial) OR XB (controlled trial) OR XB (randomised control trial) OR XB (randomization) OR XB (quasi-experimental) OR XB (pre-posttest design) OR XB (experimental study) OR XB (interventional study) | 45 |
| Cochrane Library | (Dementia):ti,ab,kw OR (Alzheimer's disease):ti,ab,kw OR (Lewy body):ti,ab,kw OR (Parkinson's dementia):ti,ab,kw OR (frontotemporal dementia):ti,ab,kw OR (mixed dementia):ti,ab,kw OR (vascular dementia):ti,ab,kw AND (cognitive stimulation therapy):ti,ab,kw OR (CST):ti,ab,kw OR (individual cognitive stimulation therapy):ti,ab,kw OR (iCST):ti,ab,kw OR (maintenance cognitive stimulation therapy):ti,ab,kw OR (MCST):ti,ab,kw OR (cognitive invention):ti,ab,kw OR (cognitive stimulation):ti,ab,kw OR (memory stimulation):ti,ab,kw OR (memory therapy):ti,ab,kw (reality orientation):ti,ab,kw OR (memory groups):ti,ab,kw OR (memory support):ti,ab,kw OR (global stimulation):ti,ab,kw OR (cognitive psychostimulation):ti,ab,kw AND (digital health):ti,ab,kw OR (digital):ti,ab,kw OR (digital medicine):ti,ab,kw OR (digital intervention):ti,ab,kw OR (digital health care):ti,ab,kw OR (electronic health):ti,ab,kw OR (ehealth):ti,ab,kw OR (mobile health):ti,ab,kw OR (mhealth):ti,ab,kw OR (technology):ti,ab,kw OR (apps):ti,ab,kw OR (applications):ti,ab,kw OR (mobile app):ti,ab,kw OR (wearable devices):ti,ab,kw OR (wearables):ti,ab,kw OR (virtual reality):ti,ab,kw OR (augmented reality):ti,ab,kw AND ("randomized controlled trial"):ti,ab,kw OR ("RCT"):ti,ab,kw OR (randomized control trial):ti,ab,kw OR (controlled trial):ti,ab,kw OR (randomised controlled trial):ti,ab,kw OR (randomization):ti,ab,kw OR (quasi-experimental):ti,ab,kw OR (pre-post test design):ti,ab,kw OR (experimental study):ti,ab,kw OR (interventional study):ti,ab,kw | 208 |
| Embase | 'dementia'/exp OR 'dementia' OR 'alzheimer disease':ti,ab,kw OR 'lewy body':ti,ab,kw OR 'parkinson dementia':ti,ab,kw OR 'frontotemporal dementia':ti,ab,kw OR 'mixed dementia':ti,ab,kw OR 'vascular dementia':ti,ab,kw AND cognitive AND stimulation AND therapy:ti,ab,kw OR cst:ti,ab,kw OR 'individual cognitive stimulation therapy':ti,ab,kw OR icst:ti,ab,kw OR 'maintenance cognitive stimulation therapy':ti,ab,kw OR mcst:ti,ab,kw OR 'cognitive intervention':ti,ab,kw OR 'cognitive stimulation':ti,ab,kw OR 'memory stimulation':ti,ab,kw OR 'memory therapy':ti,ab,kw OR 'reality orientation':ti,ab,kw OR 'memory groups':ti,ab,kw OR 'memory support':ti,ab,kw OR 'global stimulation':ti,ab,kw OR 'cognitive psychostimulation':ti,ab,kw AND 'digital health'/exp OR 'digital health' OR (digital AND ('health'/exp OR health)) OR digital:ti,ab,kw OR 'digital medicine':ti,ab,kw OR 'digital intervention':ti,ab,kw OR 'digital health care':ti,ab,kw OR 'electronic health':ti,ab,kw OR ehealth:ti,ab,kw OR 'mobile health':ti,ab,kw OR mhealth:ti,ab,kw OR 'technology':ti,ab,kw OR apps:ti,ab,kw OR applications:ti,ab,kw OR 'mobile application':ti,ab,kw OR 'wearable devices':ti,ab,kw OR 'virtual reality':ti,ab,kw OR 'augmented reality':ti,ab,kw OR 'computer platform':ti,ab,kw OR 'web based':ti,ab,kw OR 'videoconferencing':ti,ab,kw OR 'video conference':ti,ab,kw OR 'virtual meeting':ti,ab,kw OR 'zoom':ti,ab,kw OR 'google meet':ti,ab,kw AND 'randomized controlled trial'/exp OR 'randomized controlled trial' OR rct:ti,ab,kw OR 'randomized control trial':ti,ab,kw OR 'controlled trial':ti,ab,kw OR 'randomised control trial':ti,ab,kw OR randomization:ti,ab,kw OR 'quasi experimental study':ti,ab,kw OR 'pre-posttest design':ti,ab,kw OR 'experimental study':ti,ab,kw OR 'interventional study':ti,ab,kw | 126 |
| Google Scholar | Digital (computer, web-based, mobile applications, videoconferencing) cognitive stimulation therapy for dementia randomized controlled study or Digital (computer, web-based, mobile applications, videoconferencing) cognitive stimulation therapy for dementia quasi-experimental study | 5 |
| Medline (Ovid) | (Dementia or Alzheimer's disease or Lewy body or Parkinson's disease or frontotemporal dementia or mixed dementia or vascular dementia).mp. [mp=title, book title, abstract, original title, name of substance word, subject heading word, floating sub-heading word, keyword heading word, organism supplementary concept word, protocol supplementary concept word, rare disease supplementary concept word, unique identifier, synonyms, population supplementary concept word, anatomy supplementary concept word] AND (cognitive stimulation therapy or CST or individual cognitive stimulation therapy or iCST or maintenance cognitive stimulation therapy or MCST or cognitive intervention or cognitive stimulation or memory stimulation or memory therapy or reality orientation or memory groups or memory support or global stimulation or memory therapy or global stimulation or cognitive psychostimulation).mp. [mp=title, book title, abstract, original title, name of substance word, subject heading word, floating sub-heading word, keyword heading word, organism supplementary concept word, protocol supplementary concept word, rare disease supplementary concept word, unique identifier, synonyms, population supplementary concept word, anatomy supplementary concept word] AND (digital health or digital or digital medicine or digital intervention or digital health care or electronic health or ehealth or mobile health or mhealth or technology or apps or applications or mobile app or wearable devices or virtual reality or augmented reality or computer platform or web-based or videoconferencing or video conference or virtual meeting or Zoom or google meet).mp. [mp=title, book title, abstract, original title, name of substance word, subject heading word, floating sub-heading word, keyword heading word, organism supplementary concept word, protocol supplementary concept word, rare disease supplementary concept word, unique identifier, synonyms, population supplementary concept word, anatomy supplementary concept word] AND (randomized controlled trial or rct or randomized control trial or controlled trial, randomised controlled trial or randomization or quasi-experimental or pre-posttest design or experimental study or interventional study).mp. [mp=title, book title, abstract, original title, name of substance word, subject heading word, floating sub-heading word, keyword heading word, organism supplementary concept word, protocol supplementary concept word, rare disease supplementary concept word, unique identifier, synonyms, population supplementary concept word, anatomy supplementary concept word] | 31 |
| PsycInfo | ((Dementia) OR abstract(alzheimer's disease) OR abstract(Lewy body) OR abstract(parkinson's disease dementia) OR abstract(frontotemporal lobe dementia) OR abstract(mixed dementia) OR abstract(vascular dementia)) AND ((cognitive stimulation therapy) OR abstract(CST) OR abstract(individual cognitive stimulation therapy) OR abstract(iCST) OR abstract(maintenance cognitive stimulation therapy) OR abstract(MCST) OR abstract(cognitive intervention) OR abstract(cognitive stimulation) OR abstract(memory stimulation) OR abstract(memory therapy)) OR (abstract(reality orientation dementia) OR abstract(memory groups) OR abstract(memory support) OR abstract(global stimulation) OR abstract(cognitive psychostimulation)) AND ((digital health) OR abstract(digital) OR abstract(digital medicine) OR abstract(digital intervention) OR abstract(digital health care) OR abstract(electronic health) OR abstract(ehealth) OR abstract(mobile health) OR abstract(mhealth) OR abstract(technology)) OR (abstract(apps) OR abstract(applications) OR abstract(mobile app) OR abstract(wearable devices) OR abstract(virtual reality) OR abstract(augmented reality) OR abstract(computer platform) OR abstract(web-based) OR abstract(videoconferencing) OR abstract(video conference) OR abstract(virtual meeting) OR abstract(zoom) OR abstract(google meet)) AND ((randomized controlled trial) OR abstract(rct) OR abstract(randomized control trial) OR abstract(controlled trial) OR abstract(randomised controlled trial) OR abstract(randomization) OR abstract(quasi-experimental) OR abstract(pre-test post-test design) OR abstract(experimental study) OR abstract(intervention study)) | 274 |
| PubMed | ("dementia"[Title/Abstract] OR "alzheimer s disease"[Title/Abstract] OR "lewy body"[Title/Abstract] OR "parkinson s dementia"[Title/Abstract] OR "frontotemporal dementia"[Title/Abstract] OR "mixed dementia"[Title/Abstract] OR "vascular dementia"[Title/Abstract]) AND ("cognitive stimulation therapy"[Title/Abstract] OR "cst"[Title/Abstract] OR "individual cognitive stimulation therapy"[Title/Abstract] OR "iCST"[Title/Abstract] OR "maintenance cognitive stimulation therapy"[Title/Abstract] OR "mCST"[Title/Abstract] OR "cognitive intervention"[Title/Abstract] OR "cognitive stimulation"[Title/Abstract] OR "memory stimulation"[Title/Abstract] OR "memory therapy"[Title/Abstract] OR "reality orientation"[Title/Abstract] OR "memory groups"[Title/Abstract] OR "memory support"[Title/Abstract] OR "global stimulation"[Title/Abstract] OR (("cognition"[MeSH Terms] OR "cognition"[All Fields] OR "cognitions"[All Fields] OR "cognitive"[All Fields] OR "cognitively"[All Fields] OR "cognitives"[All Fields]) AND "psychostimulation"[Title/Abstract])) AND ("digital health"[Title/Abstract] OR "Digital"[Title/Abstract] OR "digital medicine"[Title/Abstract] OR "digital intervention"[Title/Abstract] OR "digital health care"[Title/Abstract] OR "electronic health"[Title/Abstract] OR "ehealth"[Title/Abstract] OR (("mobile"[All Fields] OR "mobiles"[All Fields]) AND "heatlh"[Title/Abstract]) OR "mhealth"[Title/Abstract] OR "technology"[Title/Abstract] OR "apps"[Title/Abstract] OR "mobile app"[Title/Abstract] OR "wearable devices"[Title/Abstract] OR "virtual reality"[Title/Abstract] OR "augmented reality"[Title/Abstract] OR "computer platform"[Title/Abstract] OR "web-based"[Title/Abstract] OR "videoconferencing"[Title/Abstract] OR "video conference"[Title/Abstract] OR "virtual meeting"[Title/Abstract] OR "Zoom"[Title/Abstract] OR "google meet"[Title/Abstract]) AND ("randomized controlled trial"[Title/Abstract] OR "rct"[Title/Abstract] OR "randomized control trial"[Title/Abstract] OR "controlled trial"[Title/Abstract] OR "randomised controlled trial"[Title/Abstract] OR "randomization"[Title/Abstract] OR "quasi-experimental"[Title/Abstract] OR "pre posttest design"[Title/Abstract] OR "experimental study"[Title/Abstract] OR "interventional study"[Title/Abstract])  **Translations**  **cognitive:** "cognition"[MeSH Terms] OR "cognition"[All Fields] OR "cognitions"[All Fields] OR "cognitive"[All Fields] OR "cognitively"[All Fields] OR "cognitives"[All Fields]  **mobile:** "mobile"[All Fields] OR "mobiles"[All Fields] | 22 |
| Web of Science | Dementia (Topic) or Alzheimer's disease (Topic) or Lewy body (Topic) or parkinson's dementia (Topic) or frontotemporal dementia (Topic) or mixed dementia (Topic) or vascular dementia (Topic) AND Cognitive stimulation therapy (Topic) or CST (Topic) or individual cognitive stimulation therapy (Topic) or iCST (Topic) or Maintenance cognitive stimulation therapy (Topic) or mCST (Topic) or cognitive intervention (Topic) or cognitive stimulation (Topic) or memory stimulation (Topic) or memory therapy (Topic) or reality orientation (Topic) or memory groups (Topic) or memory support (Topic) or global stimulation (Topic) or cognitive psychostimulation (Topic) AND digital health (Topic) or digital (Topic) or digital medicine (Topic) or digital intervention (Topic) or digital health care (Topic) or electronic health (Topic) or ehealth (Topic) or mobile health (Topic) or mhealth (Topic) or technology (Topic) or apps (Topic) or applications (Topic) or mobile app (Topic) or wearable devices (Topic) or virtual reality (Topic) or augmented reality (Topic) or computer platform (Topic) or web-based (Topic) or videoconferencing (Topic) or video conference (Topic) or virtual meeting (Topic) or Zoom (Topic) or Google Meet (Topic) AND randomized controlled trial (Topic) or rct (Topic) or randomized control trial (Topic) or controlled trial (Topic) or randomised controlled trial (Topic) or randomization (Topic) or quasi experimental (Topic) or pre-posttest design (Topic) or experimental design (Topic) or interventional study (Topic) | 822 |

**Appendix C**

**Risk of Bias – RoB-2**

**
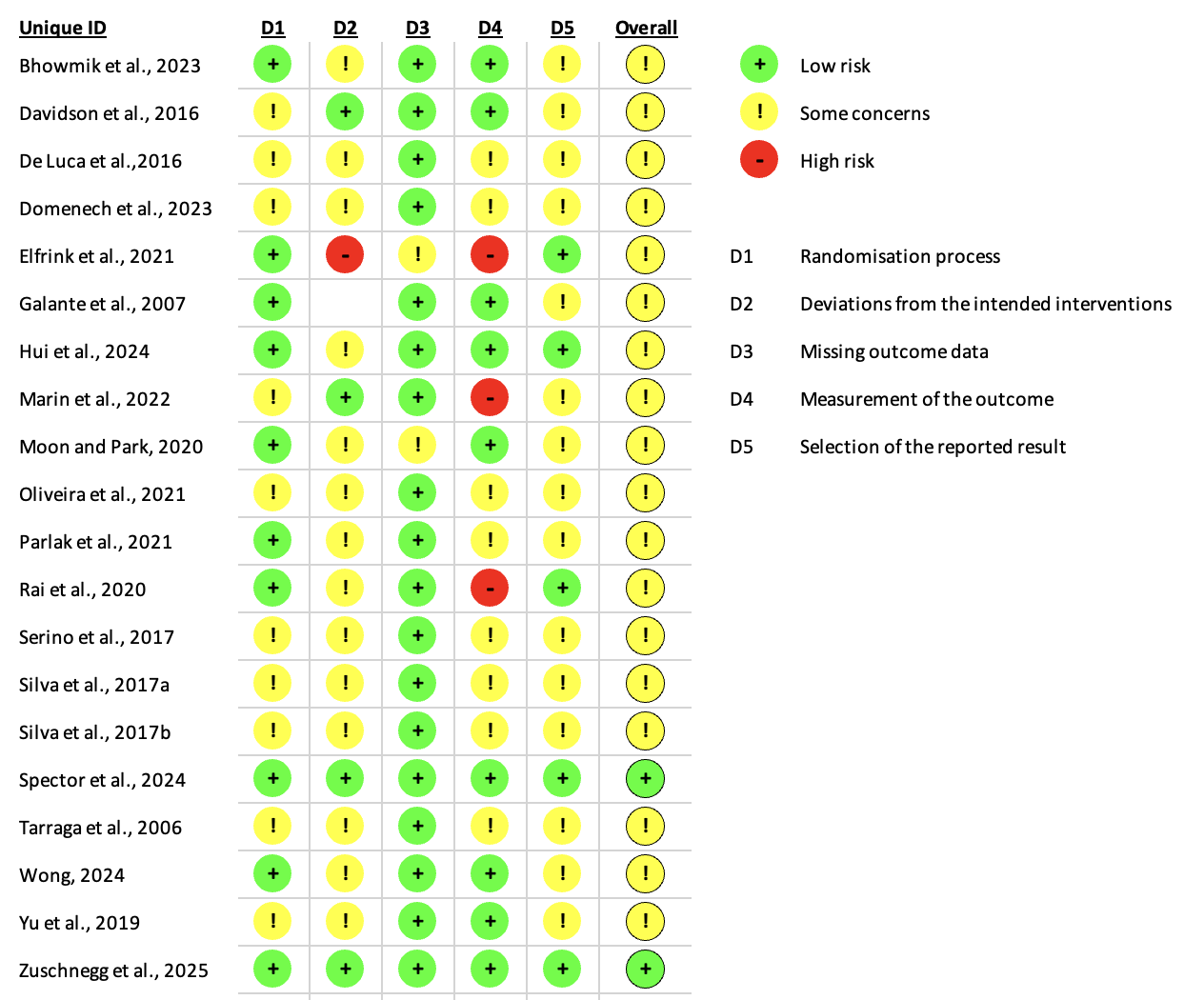
**

**Risk of Bias – ROBINS-I**

| **No** | **Domains** | **Fernández-Calvo et al., 2011** | **Gonzalez-Moreno et al., 2022** | **Sedigh et al., 2024** |
| --- | --- | --- | --- | --- |
| 1 | Bias due to confounding | L | L | L |
| 2 | Selection of participants | L | L | L |
| 3 | Clasification of interventions | L | L | L |
| 4 | Deviations from intended interventions | L | L | L |
| 5 | Missing data | L | M | M |
| 6 | Measurement outcome | L | L | L |
| 7 | Selection of the reported result | L | L | L |
| 8 | Overall bias | L | L | L |
| L, Low; M, Moderate; S, Serious; C, Critical risk of bias | | | | |
